# Clarifying Proprioceptive Training: A Narrative Review

**DOI:** 10.3390/jfmk11030279

**Published:** 2026-07-18

**Authors:** Nourali El Husseini Cardenas, Juan D. Ascuntar Viteri, Juan D. Quintero, Juan Santiago Ospina-Jimenez, Andres F. Rodriguez-Hernandez, Emmanuel Alvarez-Londoño, Jorge M. Velez, Jose A. Gomez Ortiz, Mateo Gomez-Ramirez, Andres Rojas-Jaramillo

**Affiliations:** 1 Facultad de fisioterapia, Universidad Tecnológica del Suroeste de Guanajuato, Guanajuato 36000, Mexico; hc.ibrahimel6@utsoe.edu.mx; 2 Rendimiento Físico Deportivo (RFD), Grupo de Actividad Física para la Salud (AFIS), Instituto Universitario de Educación Física y Deportes, Universidad de Antioquia, Medellín 050010, Colombia; juandavid17121997@gmail.com (J.D.A.V.); juanquintero.jdqm@gmail.com (J.D.Q.); jospina12@gmail.com (J.S.O.-J.); andres.rodriguezh06@gmail.com (A.F.R.-H.); emmanuelalvarez0915@gmail.com (E.A.-L.); arthrosgerencia@gmail.com (J.M.V.); alfrejo0101@gmail.com (J.A.G.O.); mateogomez0905@gmail.com (M.G.-R.); 3 Research Division, Dynamical Business & Science Society—DBSS International SAS, Bogotá 110311, Colombia; 4 Research Group of Sciences Applied to Physical Activity and Sport, Universidad de Antioquia, Medellín 050001, Colombia

**Keywords:** proprioception, kinaesthesia, mechanoreceptors, sensorimotor training, stroke rehabilitation, fibromyalgia

## Abstract

Proprioception is traditionally defined as the perception of joint position and movement, yet contemporary evidence supports a broader view: a multimodal sensorimotor system integrating peripheral afferent input with central motor commands. Despite its recognised importance in rehabilitation and sport, conceptual and methodological inconsistencies persist regarding its definition, assessment and training, and interventions labelled proprioceptive are frequently indistinguishable from general neuromuscular or balance-based exercise. This narrative review, reported in accordance with the Scale for the Assessment of Narrative Review Articles (SANRA), critically analyses the current state of knowledge on proprioception and clarifies the concept of proprioceptive training. PubMed, Scopus, Web of Science and SPORTDiscus were searched from January 2000 to May 2025, combining “proprioception”, “kinaesthesia”, “joint position sense” and “force sense” with “definition”, “assessment”, “training” and “rehabilitation”. Sources addressing conceptual foundations, assessment instruments, interventions explicitly labelled proprioceptive, or critical reviews were eligible, with no restriction of body region; studies on balance alone without proprioceptive measurement and non-peer-reviewed material were excluded. Of 1412 records screened, 119 informed the synthesis. Proprioception comprises distinct submodalities—joint position sense, kinaesthesia and force sense—mediated by specific receptors and pathways. Current tools isolate these under artificial conditions with limited ecological validity, and most interventions labelled proprioceptive induce general motor adaptations rather than genuine sensory ones. Submodality-specific operational definitions and ecologically valid assessment tools are required.

## 1. Introduction

Proprioception was originally termed ‘kinaesthesia’ by Henry Bastian (‘kinaesthesis’: sensation produced by movement) and redefined in 1906 by Charles Sherrington as ‘the perception of joint movement and body position in space’, referring to the fact that ‘in muscular receptivity, we see the body itself acting as a stimulus for its own receptors: the proprioceptors’ [1]. This concept has been the subject of extensive debate, leading to the view that proprioception is a sensorimotor function that, through the integration of specific peripheral sensory signals such as those from muscle spindles [2], Golgi tendon organs [3], joint and skin receptors [4,5], enables the individual to be aware, either consciously or unconsciously, of their position and movement, their perception of force and effort, and the orientation of their body or body parts in relation to their environment [2,6]. Although perceptions of the body’s position and movement have been grouped together and termed kinaesthesia, proprioception encompasses all these perceptions. Proprioception can therefore be defined as “the awareness of the mechanical and spatial state of the body and its musculoskeletal parts” [7].

Proprioception, originally conceived by Sherrington (1911) [8] as a ‘muscular sense’ focused on reflex control and postural regulation [9], marked a milestone by introducing this term to distinguish stimuli coming from the body itself from those detected by exteroceptors and interoceptors; in his foundational view, Sherrington postulated that proprioceptors, located deep in the tissues, act as sentinels that report on the state of the effectors, establishing that muscles are not only organs of execution but also of perception [10]. At the present time, this concept is defined much more completely as a fundamental multimodal sensory system that represents awareness of the mechanical and spatial state of the body and its musculoskeletal components [7], enabling the generation and constant updating of an internal body model that is indispensable for sensorimotor control and the sense of body ownership [11]. This system goes beyond the processing of peripheral feedback by integrating specific dimensions such as the sense of joint position (static), kinaesthesia or the sense of movement (dynamic), and the sense of effort or force [10], which are encoded by a diverse array of proprioceptive neurons, including Ia and II fibres from muscle spindles and Ib fibres from Golgi tendon organs [12]. Their critical role as the cornerstone of human movement lies in their capacity for dynamic sensory weighting—adjusting the relevance of signals according to the speed and reliability of the stimulus—for the control of balance [13], serving as the essential reference framework for precision and anticipation in sport, functional autonomy in daily life, and the centrepiece of rehabilitation, where the restoration of sensory acuity is vital for regaining postural stability and overcoming persistent motor deficits following an injury [14].

Research focused on proprioception spans multiple areas, ranging from anatomical and physiological foundations [15,16], neurological rehabilitation [17,18], multisensory and immersive integration, special populations [19], musculoskeletal pathologies [20,21,22,23,24,25], postural control metrics [26], effects of proprioceptive training on motor control, sporting performance and injury prevention [27,28]. Despite these advances, there are some potential inconsistencies in the understanding and application of proprioception that limit its objective application, as there is no agreed-upon definition of proprioceptive training or its programming; this can lead to confusion with other interventions that are not directly aimed at bringing about measurable neurophysiological adaptations in the sensory ability to perceive body position or movement. Furthermore, measurement instruments and techniques vary in terms of reliability and ecological validity [26,29,30], and training protocols in research studies exhibit a degree of heterogeneity that hinders the comparability and reproducibility of studies.

The clinical relevance of proprioception extends well beyond sports injury and orthopaedic rehabilitation, reaching chronic pain and neurological disorders. In fibromyalgia, altered body perception, postural instability and impaired sensorimotor integration are increasingly recognised, and proprioceptive training has been incorporated as a complementary component of multidisciplinary protocols for patients presenting with imbalance, alongside education, mindfulness and exercise [31]. This population is conceptually informative because central sensitisation and distorted body schema, rather than peripheral receptor damage, appear to drive the proprioceptive dysfunction, which raises the question of which level of the system an intervention is actually addressing.

Proprioceptive impairment is likewise one of the most frequent and functionally decisive consequences of stroke, with reported prevalence of approximately 50–64% of survivors [32,33], and it constitutes a major determinant of motor recovery, influencing gait, balance, motor learning and functional independence [32,34]. Robotic assessments have shown that sensory and motor recovery follow partly independent trajectories after stroke, so that motor gains cannot be assumed to reflect sensory restoration [35]. Contemporary neurorehabilitation therefore combines proprioceptive retraining with goal-oriented, task-specific practice [36] and with dual-task paradigms that expose cognitive–motor interference [37,38]. These clinical fields make it clear that conceptual imprecision is not merely semantic: where the therapeutic target is sensory restoration rather than motor output, mislabelling an intervention directly misdirects its design and its evaluation.

## 2. Materials and Methods

### 2.1. Justification and Objectives

This narrative review aims to clarify the concept of proprioceptive training, along with any inconsistencies in its assessment, application and effects in both clinical and sporting contexts, with a view to guiding future research.

### 2.2. Selection Criteria

Studies were included that contributed to at least one of the following areas:

(I) Conceptual foundations: studies that define proprioception, describe its physiological aspects and plausible mechanisms. (II) Assessment techniques and instruments: validation of joint position tests, detection of passive movement, etc. (III) Interventions proper: trials (randomised or non-randomised) explicitly referred to as ‘proprioceptive training’ or protocols aimed at improving proprioception. (IV) Reviews and critical analyses: narrative or systematic reviews that discuss the trainability of proprioception or highlight conceptual differences. (V) Body regions: no anatomical restriction was applied a priori. Upper-limb, trunk and cervical spine, and lower-limb districts were all eligible, and the region assessed is reported for every study in the evidence tables presented in Section 8, because receptor density, proprioceptive acuity and the reliability of the available instruments differ substantially across regions; the lower limb, and particularly the ankle and knee, nevertheless dominates the retrieved evidence and this imbalance is addressed in Section 9.1. (VI) Populations: healthy and athletic participants, musculoskeletal injury, neurological conditions in which proprioception is a recognised therapeutic target (notably stroke), and chronic pain conditions with documented sensorimotor impairment (notably fibromyalgia).

### 2.3. Exclusion Criteria

The following were excluded:

(i) Studies focusing solely on balance or postural control that were used as evidence of proprioceptive adaptation without any direct measurement of proprioception; (ii) studies in which proprioception was neither assessed nor explicitly targeted by the intervention; (iii) non-peer-reviewed sources (conference abstracts, preprints, and opinion pieces without empirical data); (iv) publications in languages other than English or Spanish; and (v) duplicate reports of a dataset already represented in the corpus. Populations with medical conditions were not excluded as a category, since neurological and chronic pain populations are precisely the settings in which proprioceptive interventions are most routinely applied; only studies in which the proprioceptive construct was absent were excluded.

### 2.4. Sources, Databases and Search Period

The search was conducted in PubMed, Scopus, Web of Science and SPORTDiscus, supplemented by backward citation tracking of the reference lists of the retrieved articles. Publications from January 2000 to May 2025 were considered for the empirical evidence base on assessment and training.

The 2000–2025 window was not intended to delimit the conceptual boundaries of the field but the body of empirical evidence on assessment and training, for three reasons. First, the instruments that dominate contemporary practice—isokinetic dynamometry with standardised joint-position-sense protocols, robotic manipulanda and stabilometric platforms—became widely available and consistently reported only from the late 1990s onwards, so earlier intervention studies are rarely comparable in measurement terms. Second, the consolidation of reporting standards for controlled trials from the mid-1990s changed the interpretability of intervention data around this boundary. Third, and most directly relevant to our aim, ‘proprioceptive training’ as an explicitly labelled intervention proliferated in the literature after 2000, and it is precisely that label whose coherence this review examines.

Critically, the date restriction was not applied to the foundational literature. Seminal pre-2000 works that established the conceptual and neurophysiological basis of proprioception were retained as mandatory references and identified through backward citation tracking, including Sherrington’s original taxonomy of proprioception [8], the classical description of joint innervation and receptor typology by Freeman and Wyke [39], and the characterisation of muscle-spindle and central contributions to position, movement and effort sense [2,10]. No account of proprioception can be sustained without them, and they are cited throughout Section 3. The restriction therefore applies to the evidence being appraised, not to the concepts being clarified.

### 2.5. Search Strategy

The following Boolean strings were applied and adapted to the syntax of each database. Block 1 (concept): (“proprioception” OR “kinaesthesia” OR “kinesthesia” OR “joint position sense” OR “force sense” OR “sense of effort”) AND (“definition” OR “concept” OR “neurophysiology” OR “mechanoreceptor” OR “muscle spindle”). Block 2 (assessment): (“proprioception” OR “kinaesthesia” OR “joint position sense” OR “force sense”) AND (“assessment” OR “measurement” OR “reliability” OR “validity” OR “threshold to detection of passive motion” OR “joint position reproduction”). Block 3 (intervention): (“proprioceptive training” OR “proprioceptive exercise” OR “sensorimotor training” OR “neuromuscular training” OR “balance training”) AND (“randomized controlled trial” OR “intervention” OR “rehabilitation” OR “athletes” OR “injury prevention” OR “stroke” OR “fibromyalgia”). Filters were limited to publication date and language; no filter was applied to study design, body region or population.

The initial search retrieved 1412 records (PubMed *n* = 486; Scopus *n* = 402; Web of Science *n* = 361; SPORTDiscus *n* = 163). After removal of 397 duplicates, 1015 titles and abstracts were screened against the criteria above and 236 full-text reports were examined. Of these, 119 sources informed the present synthesis and are cited in the reference list; 117 were excluded at full text because proprioception was neither measured nor explicitly targeted (*n* = 74), because the report was not peer-reviewed (*n* = 24), or because it duplicated a dataset already represented (*n* = 19). Screening and selection were performed independently by two authors (J.D.A. and J.D.Q.), with disagreements resolved by consensus with a third author (A.R.J.).

In direct response to the question of whether all retrieved articles were included: they were not, and by design. Consistent with the narrative nature of this review, the corpus was assembled purposively rather than exhaustively. The objective was to construct a representative and conceptually informative body of evidence sufficient to interrogate the coherence of the term ‘proprioceptive training’, not to enumerate every eligible study, which would require a systematic review with meta-analytic pooling. Where a cluster of studies shared the same design, population and outcome, representative examples were retained rather than the full set. This is a deliberate methodological choice with an unavoidable cost in reproducibility, and it is acknowledged explicitly as a limitation in Section 2.9 and Section 9.1.

### 2.6. Analysis and Categorisation

The selected studies were organized into five thematic blocks: neurophysiological foundations, assessment methods, evidence on proprioceptive training, clinical and sports applications, and inconsistencies within each block, a critical analysis of methodological quality was conducted. Findings were compared, and gaps were highlighted.

### 2.7. Structure and Contents of the Review

To make the scope of the synthesis explicit, the contents of this review are organised as follows. Section 3 addresses the neurophysiological foundations of proprioception: its definition and submodalities, the typology and distribution of joint, musculotendinous and cutaneous mechanoreceptors, the ascending afferent pathways, central integration, the interaction of feedforward and feedback control, and the neuroplasticity of the system across training, ageing and injury. Section 6 examines the validated techniques and instruments available for assessing proprioception and compares their psychometric performance, the submodality each one isolates, and their ecological validity. Section 7 analyses the application of the construct in sport, including injury-prevention claims and the use of external aids. Section 8 presents the evidence retrieved on proprioceptive training in rehabilitation and readaptation (Section 8.1.1) and in injury prevention and sports performance (Section 8.1.2), with the load parameters of each protocol tabulated. Section 9 discusses the conceptual and methodological inconsistencies identified, with specific attention to neurological rehabilitation and chronic pain, and Section 10 states the resulting conclusions.

Two boundaries of scope should be stated. First, this review is concerned with the coherence of proprioceptive training as a construct, not with quantifying its efficacy; effect sizes are therefore reported only where they bear on the conceptual argument. Second, the review addresses proprioception as a sensory and sensorimotor construct, and consequently does not cover vestibular or visual rehabilitation except where these systems interact with proprioceptive weighting.

### 2.8. Registration, Transparency and Data Availability

This narrative review was not eligible for registration in PROSPERO, which restricts registration to systematic reviews with health-related outcomes and explicitly excludes narrative and scoping reviews. To address the underlying concern about methodological consistency and transparency, the review protocol was instead documented and deposited in an open repository (Figshare), comprising the research question and objectives, the eligibility criteria, the full Boolean strings per database with each date executed, the record-level screening decisions with reasons for exclusion, the data-extraction sheet underlying the evidence tables, and the SANRA self-assessment described in Section 2.9.

Repository record: Figshare, https://doi.org/10.6084/m9.figshare.32101657 (publicly accessible, accessed on 15 July 2026). The record has been released so that the eligibility criteria, the search strings and the selection process reported in Section 2.2, Section 2.3, Section 2.4 and Section 2.5 can be inspected and audited independently by editors, reviewers and readers.

### 2.9. Methodological Limitations

As this review was not systematically protocolised, it may exhibit selection bias and methodological heterogeneity that preclude meta-analysis. To mitigate these risks, the review was prepared and reported in accordance with the Scale for the Assessment of Narrative Review Articles (SANRA) [40], a six-item instrument covering the justification of the article’s importance, the statement of concrete aims, the description of the literature search, referencing, the presentation of evidence levels, and the appropriate presentation of data. Each item was addressed explicitly: the importance and aims are stated in Section 1 and Section 2.1; the search is described in Section 2.4 and Section 2.5; evidence levels and the methodological quality of each protocol are appraised critically within every thematic block rather than pooled; and the load parameters of the retrieved interventions are presented in the evidence tables in Section 8 so that readers can verify the basis of our claims. Two authors independently rated the manuscript against SANRA and the completed self-assessment has been deposited with the protocol (Section 2.8). SANRA does not eliminate selection bias, and we do not claim that it does; it makes the basis of our selection auditable.

## 3. Physiological Aspects and Plausible Mechanisms Associated with Proprioception

### 3.1. Definition and Dimensions of Proprioception

To place proprioception within its proper neurophysiological context, it is essential to start with Sherrington’s foundational taxonomy (1906) [8], who categorised the organism’s sensory experience according to the spatial origin of the stimulus: exteroception, responsible for processing information from the external environment (such as vision, hearing or superficial touch); interoception, responsible for monitoring the visceral and homeostatic environment (internal organs); and proprioception, originating in deep tissues to provide information on the mechanical and spatial state of the body itself [9].

However, contemporary scientific reviews have drastically expanded this classical notion of a simple peripheral ‘muscle sense’. Today, proprioception is reconceptualised as a high-level multimodal system that enables the generation and continuous updating of an internal body model, ensuring a sense of body ownership through the constant integration of peripheral afferents with central motor commands [7,11].

Within this updated framework, the proprioceptive system operates through three classic functional dimensions, each exhibiting clear molecular, anatomical and neurophysiological specialisation [12].

#### 3.1.1. Joint Position Sense (Static and Dynamic)

This dimension defines the nervous system’s ability to perceive the three-dimensional location of a body segment, both at rest (identification of a static joint angle) and during the course of a motor task (dynamic perception of spatial relationships). It is predominantly mediated by afferents from type II muscle spindle fibres, in conjunction with capsular joint receptors (such as Ruffini corpuscles). Its primary function is to provide the somatosensory mapping necessary for the brain to determine the exact configuration of the limbs before, during and after motor execution [10].

#### 3.1.2. Kinaesthesia or Sense of Movement

Unlike the sense of position, kinaesthesia focuses specifically on the perception of speed, acceleration and the direction of joint movement. It is primarily encoded by the fast-adapting type Ia fibres of the muscle spindles and, secondarily, by the joint Pacini corpuscles. Given its extremely high sensitivity to variations in dynamic stretch, this dimension is critical for sensory reweighting mechanisms and rapid anticipatory correction, enabling the nervous system to adjust balance control in response to external disturbances within milliseconds [12,13].

#### 3.1.3. Sense of Effort or Muscle Strength

**Figure 1:**
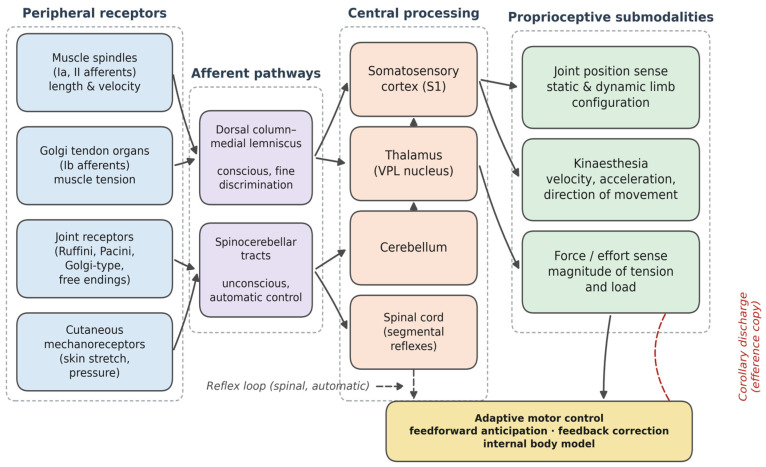
Conceptual model of proprioception: from peripheral receptors to motor output. Solid arrows indicate the direction of afferent signal flow from receptors to central structures and onward to the proprioceptive submodalities; the red dashed line indicates corollary discharge (efference copy) from motor commands; the grey dashed arrow indicates the spinal reflex loop, which bypasses conscious processing.

This encompasses the ability to perceive, quantify and modulate the muscle tension generated, as well as the magnitude of an external mechanical load or resistance. Although the tension stimulus is transduced at the cell periphery by the Ib fibres of the Golgi tendon organs, its conscious perception and central modulation depend strictly on integration with the corollary discharge (an efferent copy of the motor command sent from the motor cortex to the cortical sensory areas). It is this bidirectional interaction that allows the magnitude of the effort exerted to be precisely calibrated in relation to peripheral fatigue or the changing demands of the environment [7,10]. To facilitate the understanding of proprioception as a multimodal sensorimotor system, a conceptual model integrating peripheral receptors, afferent pathways, central processing, and functional submodalities is presented in Figure 1.

The figure illustrates the integration of peripheral mechanoreceptors (muscle spindles, Golgi tendon organs, joint and cutaneous receptors), afferent pathways (spinocerebellar and dorsal column–medial lemniscus systems), and central processing (cerebellum, thalamus, and somatosensory cortex), leading to the emergence of proprioceptive submodalities (joint position sense, kinaesthesia, and force perception) and their role in adaptive motor control.

### 3.2. Mechanoreceptors: Types and Specific Function

#### 3.2.1. Joint Mechanoreceptors

To complete the peripheral somatosensory map and complement intramuscular mechanotransduction, it is essential to analyse the role of joint mechanoreceptors. Although their functional significance relative to muscle spindles has historically been debated, current evidence confirms that these receptors act as a critical system for early warning, reflex stabilisation and modulation of motor control, operating synergistically with muscle afferents.

According to the classical neuroanatomical classification (Freeman and Wyke) [39], these mechanoreceptors are divided into four main types, each with specific neurophysiological properties and activation thresholds:

##### Ruffini Corpuscles (Type I)

These are the most abundant joint receptors and are characterised by a low threshold and slow adaptation. This physiological configuration allows them to be activated by minimal mechanical stimuli and to continue firing action potentials in a sustained manner whilst the deformation persists.

Physiological Function: They respond primarily to changes in static position and direction of movement, and are exquisitely sensitive to changes in joint capsule tension. Their continuous feedback is essential for maintaining basal muscle tone via monosynaptic reflexes, ensuring static postural stability.

##### Pacini Corpuscles (Type II)

These encapsulated receptors have a low threshold but adapt rapidly. This means they fire electrical signals transiently, only in response to the onset, cessation, or rapid changes in a mechanical stimulus.

Physiological Function: They are functionally ‘blind’ to static position; their speciality is the detection of acceleration, deceleration and mechanical vibration. They are indispensable to the central nervous system during rapid dynamic phases, such as the transition in the gait cycle or in explosive sporting movements, providing information on sudden changes in joint inertia.

##### Articular Golgi-Type Corpuscles (Type III)

Structurally identical to the tendinous Golgi organs, but located in the connective tissue of the joint, these receptors have a high threshold and a slow adaptation.

Physiological Function: They remain silent for most of the free range of motion (ROM). They are activated exclusively at the extremes of the ROM or when the joint is subjected to high longitudinal tension (traction). Upon activation, they trigger a powerful reflex protection mechanism: they inhibit the agonist muscles and facilitate the contraction of the antagonist to stabilise the joint and prevent imminent structural damage.

##### Free Nerve Endings (Type IV)

These constitute the extensive network of joint nociceptors. They are fine, unmyelinated nerve fibres, characterised by a very high threshold and no adaptation.

Physiological Function: Under conditions of tissue integrity, they remain inactive. They are activated exclusively in pathological or extreme conditions, such as severe mechanical deformation, local ischaemia or chemical irritation caused by inflammatory mediators. Their activation not only mediates the perception of pain, but also triggers arthrogenic muscle inhibition, a spinal reflex that temporarily ‘shuts down’ the surrounding muscles to immobilise and protect the injured area.

##### Density and Anatomical Distribution

The network of joint mechanoreceptors is not distributed uniformly or homogeneously; its topography is strictly determined by the biomechanical and motor control demands of each body region:

Proximal–distal gradient: There is a density of receptors that is inversely proportional to the size of the joint. Distal joints (hands, feet and ankles) exhibit a significantly higher density than proximal joints (hip, shoulder). This high peripheral concentration responds to the need to make constant micro-postural adjustments and to facilitate fine motor skills during direct interaction with the environment.

Cervical Region: The facet joints of the upper cervical spine (C1–C3) exhibit an exceptionally high concentration of receptors (particularly Type I). This extreme density is neurophysiologically crucial, as it enables the rapid integration of cervical proprioception with visual and vestibular inputs, forming the central circuit for the control of balance and spatial orientation.

Intra-articular Tissue Topography: The location of the receptors is highly specific. Type I (Ruffini) and Type IV (Free) receptors predominate in the superficial layers of the fibrous capsule. Type II (Pacini) receptors are clustered in the deep capsular layers and, in abundance, in the intra-articular fat pads. Type III (articular Golgi) receptors are located almost exclusively in the matrix of the intrinsic and extrinsic ligaments. It is essential to note that tissues subjected to constant friction and compressive load, such as hyaline cartilage and the synovial membrane, are completely devoid of mechanosensitive or nociceptive innervation [10,14,39].

#### 3.2.2. Musculotendinous Receptors

To understand the mechanotransduction basis of the proprioceptive system, it is essential to analyse the physiology of the two main intramuscular mechanoreceptors: the muscle spindle and the tendinous organ of Golgi (TOG). Both operate according to opposing yet complementary mechanical principles, forming a closed-loop feedback system that is indispensable for motor control [9,12].

##### Muscle Spindles: Sensors of Length and Speed

Located in the muscle belly and arranged parallel to the extrafusal fibres (which are responsible for motor contraction), muscle spindles are encapsulated structures specialised in detecting tissue stretching.

Intrafusal Fibres and Sensory Afferents (Ia and II): The interior of the receptor contains infrafusal fibres innervated by two types of terminals. Type Ia (primary) fibres are fast-conducting neurons that primarily detect the rate of change in muscle length, forming the mechanotransducing basis of kinesthesia. Type II (secondary) fibres, on the other hand, are slow-adapting and respond strictly to the static length of the muscle, making them fundamental for the sense of joint position [10].

Gamma (γ) Efferent Innervation and Co-activation: Unlike other receptors, the muscle spindle has its own motor innervation. To prevent the spindle from becoming ‘slack’ and silent during muscle contraction, the central nervous system utilises alpha–gamma co-activation: whilst the muscle shortens (via alpha motor neurons), gamma motor neurons contract the ends of the intrafusal fibres, keeping the receptor under mechanical tension and highly sensitive throughout the range of motion.

Stretch (Myotatic) Reflex: This constitutes the fastest monosynaptic circuit in the nervous system. In response to a sudden stretch, Ia fibres fire an action potential that travels to the spinal cord and directly excites the alpha motor neuron of the same muscle, causing its immediate contraction to oppose the elongation and stabilise posture [9].

##### Golgi Tendon Organs (GTOs): Tension Sensors

Located at the myotendinous junction, GTOs are encapsulated collagen networks arranged in series with respect to the extrafusal muscle fibres, functioning physiologically as mechanical force sensors.

Mechanical Transduction (Ib Fibres): The GTO is innervated by thick, myelinated Ib-type afferents. When the tendon is under tension, the collagen strands compress, ‘squeezing’ the nerve ending and opening mechanosensitive ion channels that trigger the electrical signal [12].

Active vs. Passive Tension Response: The OTG is exquisitely sensitive to active tension; that is, it requires very little force to activate when it is the muscle itself that contracts and pulls on the tendon. Conversely, it has a markedly higher activation threshold in response to passive tension (when an external agent stretches the musculotendinous complex).

Protective Function (Inverse Myotatic Reflex): In the face of extreme mechanical overload or a contraction that threatens the structural integrity of the tendon, the OTG triggers high-frequency signals. In the spinal cord, Ib fibres activate inhibitory interneurons that suppress the activity of the alpha motor neuron of the agonist muscle, causing its sudden relaxation and preventing tears or avulsions.

##### Systemic Interaction: Regulation of Muscle Tone

Basal muscle tone (the passive, preparatory stiffness of a muscle at rest) is not an inert property, but a neurological state finely regulated by the balance between these two peripheral systems.

This regulation operates under a delicate system of checks and balances: gravity and postural micro-movements constantly stretch the muscles, causing the muscle spindles to send continuous excitatory signals (facilitatory tone) to maintain an upright posture. Simultaneously, as muscle tension increases due to this contraction, the OTGs send modulatory inhibitory signals that prevent activity from escalating into pathological rigidity (such as spasticity). The central nervous system orchestrates this balance by modulating the ‘gain’ of gamma motor neurons, thereby adjusting the sensitivity of the entire system to the specific demands of the environment.

#### 3.2.3. Cutaneous Receptors with Proprioceptive Function

To complete the picture of peripheral mechanotransduction, it is crucial to break down the traditional boundary that isolates the skin as a purely exteroceptive organ. Modern neurophysiological literature [7,10] demonstrates that cutaneous mechanoreceptors are, in fact, indispensable components of the proprioceptive system, providing critical information for the construction of the ‘internal body model’ and fine motor control.

The role of these receptors, their specialised topography and their profound clinical implications are detailed below:

##### Cutaneous Mechanoreceptors and Their Proprioceptive Contribution

The skin, particularly in the areas adjacent to the joints, acts as an enveloping biomechanical sensor. When a joint moves, the skin on one side is stretched and that on the opposite side is compressed, generating a pattern of signals that the brain decodes as joint movement.

Dermal Ruffini corpuscles (Slow Adaptation II): These are extremely sensitive to directional stretching of the skin. Their feedback is vital for joint position sense (JPS) and kinaesthesia, particularly in distal joints where the muscle belly is far from the joint being moved (e.g., the fingers).

Merkel discs (Slow Adaptation I): These respond to sustained pressure and spatial deformation. They are fundamental for encoding the magnitude of the mechanical load borne by a body surface against gravity or when manipulating an object.

Meissner corpuscles (Fast Adaptation I): These are detectors of micro-movements and friction. In the proprioceptive context, they provide information on the acceleration of contact and are essential for detecting the ‘slip’ of an object in the hand or a change in inertia under the foot.

##### Topographical Specialisation: Palms, Soles and Fingers

The skin’s contribution to proprioception follows a distal gradient. On the hairless surfaces of the hands and feet, the density of these receptors is extraordinarily high, making them the highest-resolution ‘interfaces’ between the body and the environment.

Hand and Fingers (Manipulation): In the hand, the information provided by the muscle receptors in the forearm is insufficient for tasks requiring fine motor skills. The brain relies on the stretching of the skin on the back of the hand (Ruffini) to know exactly how many degrees a finger has been flexed, and on the pressure in the fingertips (Merkel/Meissner) to modulate grip strength (force matching) without breaking the object or dropping it.

Sole of the Foot (Postural Stability): The sole of the foot functions as a dynamic pressure matrix. Plantar cutaneous mechanoreceptors constantly map the distribution of the centre of pressure (CoP) and the texture of the ground. This ‘exteroceptive proprioception’ is the primary input for the balance control circuits before the disturbance reaches the ankles or knees.

##### Clinical Relevance: Rehabilitation and Footwear

Understanding the skin as a proprioceptive organ transforms clinical and sports intervention strategies:

Rehabilitation and Sensory Reweighing: In patients with ligament damage (e.g., functional ankle instability [14], the brain increases its reliance on plantar skin signals and the stretching of the skin around the ankle to compensate for the deficit in deep mechanoreceptors and maintain balance [13]. Modern therapies use functional taping not only to provide mechanical restriction, but also to artificially increase skin traction and ‘stimulate’ Ruffini receptors, thereby improving joint awareness.

The interaction between the foot and the ground is mediated by footwear, which acts as a ‘sensory filter’. Footwear with excessively thick, rigid or super-cushioned soles severely attenuates mechanical deformation and filters out vibration frequencies. This silences Merkel’s discs and Pacini/Meissner corpuscles, depriving the nervous system of ‘low-level’ proprioceptive information [7]. This explains why chronic use of hyper-cushioned footwear can degrade plantar proprioceptive acuity, altering gait and increasing the risk of falls in older adults, whilst barefoot or minimalist footwear training is used to recalibrate this somatosensory mappara recalibrar este mapa somatosensorial.

### 3.3. Ascending Afferent Pathways

Once mechanical stimuli are transduced by peripheral receptors, the information must travel to the central nervous system (CNS) via high-fidelity afferent networks. For the ‘internal body model’ to be updated in real time and enable motor correction [11], proprioceptive transmission relies on the body’s thickest and fastest nerve fibres, channelled through three main ascending pathways.

#### 3.3.1. Fibre Types and Conduction Velocities

The efficiency of these pathways is determined by the degree of myelination and the diameter of the afferent axons [12,41]:

Type Ia and Ib fibres (motor classification)/Aα\alpha fibres (sensory classification): These innervate muscle spindles (dynamic) and Golgi tendon organs. They have the largest diameter (12–20 µm) and the highest conduction velocity in the human body, ranging from 70 to 120 m/s. They are essential for ultra-rapid reflex adjustment [12,41] Type II fibres/Aβ\beta fibres: they innervate the secondary spindle endings (static), the joint mechanoreceptors (Ruffini, Pacini) and skin mechanoreceptors (Merkel, Meissner). They have a moderate diameter (6–12 µm) and a fast conduction velocity, ranging from 30 to 70 m/s, which is fundamental for conscious proprioception and tactile discrimination [41].

At the spinal and supraspinal levels, these afferents are distributed across three parallel tracts with highly specialised functions:

#### 3.3.2. Spinocerebellar Pathway: Automatic Control

This pathway is primarily responsible for unconscious or ‘low-level’ proprioception [7]. It transmits massive and continuous information on muscle length and tension, as well as joint dynamics, directly from the spinal cord to the cerebellum [9]. Anatomy and Transmission: Unlike other pathways, spinocerebellar information (via the dorsal and ventral spinocerebellar tracts and the cuneocerebellar tract) is projected predominantly ipsilaterally; that is, the right cerebellar hemisphere receives and integrates information from the right side of the body, without decussation in the brainstem [41].

Physiological Function: The cerebellum uses this incessant flow of spatial and biomechanical data to compare ‘motor intention’ (corollary discharge from the cortex) with ‘actual performance’ (peripheral feedback). This pathway forms the basis of automatic motor control, the subconscious coordination of gait and anticipatory postural adjustments, operating at speeds beyond conscious perception [9,10].

#### 3.3.3. Dorsal Column-Medial Lemniscus (DCML) Pathway: Conscious and Fine Proprioception

This is the ‘major circuitry’ for high-level proprioception [7], kinaesthesia, position sense discrimination and vibration perception (Pacini corpuscles).

Anatomy and Transmission: The Aα\alpha and Aβ\beta fibres enter the spinal cord and ascend ipsilaterally via the dorsal columns (gracile fascicle for the lower limbs and cuneiform fascicle for the upper limbs). Upon reaching the medulla oblongata, they synapse in the gracile and cuneiform nuclei, decussate (cross the midline) and ascend to form the medial lemniscus [41].

Central Integration: The pathway culminates by forming synapses in the ventrolateral posterior nucleus (VPL) of the thalamus, the major sensory relay centre, from where it projects to the primary somatosensory cortex (S1) in the parietal lobe.

Physiological Function: This pathway enables the cortical representation of the somatosensory map [10]. It is essential for the individual to consciously recognise the angle of their joints, discriminate the weight of objects, and experience a sense of body ownership [11].

#### 3.3.4. Spinothalamic Tract: Sensory-Motor Integration and Nociception

Historically described as the main pathway for pain and temperature, the spinothalamic tract (which carries signals from slower A\delta and C fibres, innervating Type IV free nerve endings) plays an irreplaceable integrative role in the context of injury and rehabilitation.

Anatomy and Transmission: Afferents enter via the dorsal horn of the spinal cord, synapse almost immediately, decussate at the spinal segment level, and ascend through the anterolateral region towards the thalamus and multiple cortical areas, including the limbic system [41].

Proprioceptive Relevance: Proprioception does not occur in a mechanical vacuum. In the presence of joint pathology, trauma or ischaemia, the spinothalamic tract is intensely activated. At the spinal cord level, this nociception interacts with proprioceptive circuits, triggering arthrogenic muscle inhibition and altering motor commands to protect the structure. At the cortical level, the integration of nociceptive and proprioceptive signals modifies the conscious body schema, explaining why painful movements or fear of movement (kinesiophobia) dramatically disrupt high-level proprioceptive acuity and postural stability [7,14].

Efficient motor control depends on the integrity and simultaneous processing of these three pathways, merging the immediacy of spinal reflexes and cerebellar coordination with the sophistication of the cortical somatosensory map.

### 3.4. Central Integration and Sensorimotor Processing

The processing of proprioceptive information does not constitute a simple passive relay pathway, but rather a hierarchical and parallel integration system along the rostrocaudal axis of the central nervous system (CNS). As afferent signals ascend, the representation of the ‘internal body model’ becomes progressively more complex, moving from local reflex control to conscious perception and abstract motor planning [11].

This processing is fundamentally orchestrated across three levels of integration:

#### 3.4.1. Spinal Cord Level: The Segmental Reflex Response

The spinal cord is the first processing station and acts as a ‘low-level’ control centre designed for immediacy. Here, proprioceptive integration occurs within milliseconds without conscious intervention, and is critical for the structural survival of tissues and the maintenance of basal tone [9].

Monosynaptic Arcs: Ultra-fast afferents (Ia fibres from muscle spindles) enter via the dorsal horn and establish direct excitatory synapses with the alpha motor neurons of the corresponding muscle. This forms the basis of the myotatic or stretch reflex, which enables an immediate motor correction in response to unexpected disturbances.

Polysynaptic circuits: Other afferents, such as the Ib fibres from the Golgi tendon organs, branch off into networks of inhibitory and excitatory interneurons. These polysynaptic circuits regulate the inverse myotatic reflex (protection against overload) and reciprocal inhibition (relaxation of the antagonist during contraction of the agonist), forming the basis of primary muscle synergy.

#### 3.4.2. Brainstem and Cerebellum: Motor and Postural Refinement

Via the spinocerebellar tract, the cerebellum and brainstem nuclei (such as the vestibular nuclei and the reticular formation) receive a massive and continuous flow of unconscious proprioception.

Coordination and Balance: The cerebellum operates as a powerful signal processor and error comparator. It receives the ‘motor intention’ from the cerebral cortex (corollary discharge) and compares it in real time with the ‘actual mechanical performance’ reported by peripheral proprioceptors [10].

Automatic Postural Adjustments (APA): If there is a spatial or temporal discrepancy, the cerebellum projects compensatory signals to the brainstem and spinal cord to fine-tune the ongoing movement. This network is indispensable for sensory reweighting [13], rapidly adjusting the centre of gravity and stabilising posture before the conscious brain perceives the instability. 

Cerebral Cortex and Basal Ganglia: ‘Higher-Level’ Sensory-Motor Control

Information ascending via the medial lemniscus reaches the thalamus and radiates out to the telencephalic circuits, where mechanical sensation is transformed into perception and voluntary action [7].

Primary Somatosensory Cortex (S1): Located in the postcentral gyrus, it receives direct afferents to construct a precise somatotopic map (the sensory homunculus). S1 is responsible for fine discrimination: it consciously decodes the exact joint position, segment velocity and magnitude of muscle tension.

Secondary Somatosensory Cortex (S2) and Posterior Parietal Cortex: These areas act as centres of multimodal integration. They fuse the mapping from S1 with visual and vestibular information to consolidate body ownership and update the dynamic internal body model in three-dimensional space [11].

Motor Areas (M1, Premotor and SMA): The motor cortex cannot function in a vacuum; it requires the spatial coordinates provided by the somatosensory areas. The Supplementary Motor Area (SMA) and the Premotor Cortex use this updated body schema to plan complex, anticipatory movement sequences. Finally, the Primary Motor Cortex (M1) executes the command, sending the descending signal to the spinal cord and, simultaneously, issuing a new corollary discharge that restarts the feedback cycle.

The Integrative Role of the Basal Ganglia: Working in close collaboration with the motor cortex, the basal ganglia process proprioceptive context for the selection of motor programmes. They act as a ‘gate’: they facilitate the execution of desired and automated motor plans (learned and fluid movements) whilst actively suppressing competing or unwanted muscle commands, ensuring clean and efficient motor control.

### 3.5. Motor Control: Feedforward and Feedback Systems

Motor execution is not limited to mere muscle contraction; it demands impeccable temporal coordination between prediction and reaction. To achieve the fluidity and precision that characterise human movement, the central nervous system (CNS) simultaneously employs two sensorimotor control strategies that, although mechanistically distinct, are functionally inseparable:

#### 3.5.1. Feedforward (Anticipatory) Control

This mode of control is intrinsically proactive and predictive. Rather than reacting to a peripheral stimulus, the CNS uses prior motor experience stored in the cerebral cortex and cerebellum to generate a prediction of the mechanical demands of the task to be performed.

Mechanism: This is based on the activation of prior internal body models [11]. The brain sends the motor command to the effectors without needing to wait for an active afferent signal or peripheral feedback to validate the action.

Physiological Advantage: Its main virtue is extreme speed. As it does not depend on the anatomical conduction time of sensory pathways or synaptic delays, it allows stabilising responses to be generated before the mechanical disturbance occurs.

Clinical Example: Anticipatory Postural Adjustments (APA). Milliseconds before a person lifts a heavy object with their arms, the CNS pre-activates the stabilising muscles of the ankle, leg and trunk. This anticipation ‘anchors’ the body to the ground and prevents the sudden shift in the centre of mass from causing the individual to lose balance towards the side.

#### 3.5.2. Feedback Control (Corrective)

Unlike the anticipatory system, feedback control is reactive in nature and structurally depends on the closed-loop sensorimotor system.

Mechanism: This relies entirely on real-time proprioceptive signals. Muscle spindles, Golgi tendon organs, and mechanoreceptors provide continuous feedback on the body’s mechanical state. Structures such as the cerebellum compare this peripheral input with the original “motor intention” to calculate the margin of error [10].

Physiological Advantage: Although it is an inherently slower system—due to the need to convey the stimulus to the CNS and send the response back to the muscle—it is vastly more adaptive and precise.

Clinical Example: This control is the essential rescue mechanism when walking on uneven terrain, when facing unexpected disturbances (such as slipping), or when learning novel movements where the “internal model” does not yet have sufficient experience to predict the outcome.

#### 3.5.3. Coexistence and Functional Complementarity

In the biomechanics of daily life and sports, the separation of these systems is purely theoretical, as they coexist and complement each other inseparably in every motor gesture. Feedforward control orchestrates the rapid and fluid initiation of movement based on a calculated prediction, while feedback control simultaneously acts as a dynamic safety net: it monitors execution and corrects on the fly any micrometric discrepancy between what the brain anticipated and what the reality of the environment demanded.

### 3.6. Neuroplasticity and Adaptation of the Proprioceptive System

The proprioceptive system is neither a static network of wiring nor immutable ‘hardware’; it constitutes a highly dynamic neurobiological matrix that is continually modified in response to mechanical demands and experience. This capacity for bidirectional adaptation—which allows both the refinement of sporting movements and compensation following a deficit—is the biological foundation that justifies any clinical or sporting intervention.

The following section explores how proprioception adapts in different physiological and clinical scenarios, with the plasticity of the central nervous system (CNS) as the central focus.

#### 3.6.1. Proprioceptive Training and Motor Learning

During the acquisition of a new motor skill or in high-performance sports training, the CNS not only hypertrophies the muscle but also ‘refines’ its afferent pathways.

Specific training induces an improvement in the resolution of the internal body model [11].

The system learns to perform more efficient sensory weighting [13], rapidly adjusting which peripheral signals (e.g., muscle spindles versus vision) are most reliable depending on the speed and context of the movement. This results in shorter response latency, greater anticipation (feedforward control) and more economical and precise motor execution.

#### 3.6.2. Ageing (Sensory-Motor Senescence)

The life cycle imposes anatomical and functional deterioration on the system. With ageing, there is a decrease in the number and sensitivity of peripheral mechanoreceptors, as well as a slowing of the conduction velocity of large nerve fibres [42].

This decline impairs ‘low-level’ proprioception, forcing older adults to rely excessively on vision to maintain balance. However, evidence shows that specific training in older adults can mitigate this loss, promoting synaptic reorganisations that partially restore proprioceptive acuity and prevent the risk of falls [42].

## 4. Recovery Following Injury (Deficit and Compensation)

When tissue damage occurs, such as in functional ankle instability (FAI), not only is joint biomechanics altered, but the flow of information from the capsuloligamentous mechanoreceptors is also disrupted.

This peripheral deafferentation ‘blurs’ the somatosensory map, which directly contributes to a persistent alteration in postural stability, even when gross muscle strength has recovered [14].

The system attempts to compensate for this deficit by shifting the weight of information towards uninjured mechanoreceptors (such as those in the skin or proximal joints), a process that often results in aberrant motor patterns or secondary overloads if not therapeutically guided.

## 5. Cortical Plasticity: The Underlying Mechanism

The biological substrate that enables all these modifications—learning, compensation and recovery—is neuroplasticity, particularly at the level of the cerebral cortex.

The Primary Somatosensory Cortex (S1) and parietal integration areas are not rigid maps; they are dynamic. Repetition of a stimulus (training) expands the cortical representation (homunculus) of the joint involved, increasing synaptic density and the capacity for fine discrimination.

Conversely, disuse, immobilisation or peripheral damage causes a contraction of that cortical representation, a phenomenon known as ‘sensorimotor amnesia’ [7].

Plasticity allows the brain to continually rewrite the sense of body ownership to adapt to the body’s new mechanical reality [11].

### Conclusions: Justification for Rehabilitation Intervention

This profound capacity for modification directly links physiological mechanisms with clinical practice. It conclusively demonstrates that physical rehabilitation and sports rehabilitation are not purely orthopaedic or biomechanical processes, but profoundly neurological interventions.

## 6. What Are the Validated Techniques and Instruments for Assessing Proprioception?

To properly answer the question of what techniques and instruments are available for assessing proprioception, it must first be made clear that this ability cannot be understood as a single or uniform phenomenon. Studies such as those by Hillier and Immink [43] suggest that it should be approached as a set of sub-modalities, namely the sense of joint position, the sense of movement (kinesthesia) and the sense of force or effort. These domains are neither equivalent nor interchangeable, which has direct implications for the instruments used. For example, a test such as the Threshold to Detection of Passive Motion, which assesses the threshold at which passive joint movement is detected, is useful for evaluating the sense of movement, but cannot be extrapolated to the perception of force or static position, as it primarily stimulates mechanoreceptors linked to movement. This lack of specificity has led to significant methodological variation in the literature, where many tasks with a sensorimotor component are labelled as proprioception tests, without specifying which submodality is actually being assessed [44].

One of the most commonly used strategies is the active reproduction of joint position, in both clinical and experimental contexts. In this task, the subject must adopt a position and then voluntarily reproduce it without visual cues. Although it allows for the assessment of the sense of position, it also introduces cognitive and motor variables that may interfere with its specificity, such as working memory and motor control [7]. Similarly, Röijezon and Clark [45] note that factors such as pain or fatigue can compromise the reliability of repositioning tests, particularly in regions such as the cervical and lumbar spine. Another common technique is the detection of the passive movement threshold, which has the advantage of reducing motor influence, although it requires precise equipment and standardised protocols [7]. In contrast, clinical tools such as the Rivermead Assessment of Somatosensory Perception, although less sensitive, prove useful in clinical settings and in populations with neurological damage [43].

With technological advances, new instruments have emerged to address different dimensions of proprioception. For example, robotic manipulators allow passive movement thresholds to be detected with high precision, focusing on kinesthesia [44]. Stabilometric platforms, such as the Pro-Kin system, assess proprioceptive accuracy in balance tasks from a more integrative approach [26]. And cervical repositioning systems quantify angular errors in people with chronic pain [45]. However, these tools have significant limitations, being highly complex, of limited clinical applicability, and difficult to use for isolating proprioceptive submodalities.

From a critical perspective, Héroux and Butler [7] caution that many tests are limited to low-level proprioceptive judgements and do not capture sensory integration in dynamic environments. Thus, demonstrating accuracy in static laboratory tests does not guarantee that the proprioceptive system will respond effectively to more complex functional demands. Clark and Röijezon [44] agree that many assessments are conducted under artificial conditions and do not reflect the functional role of proprioception in motor behaviour. In line with this, Röijezon and Clark [44] emphasise that proprioception must be analysed within the framework of its interaction with motor response and the physical environment, as this integration is important for performance in everyday activities.

The methodological limitations of the reviewed studies should also be highlighted. Some studies, such as that by Justo-Cousiño and Da Cuña-Carrera [46], employ randomised placebo-controlled designs; however, their conclusions regarding the efficacy of neuromuscular taping or Kinesio Taping in improving proprioception are tempered by the fact that they assessed healthy subjects with no prior proprioceptive deficits. Similarly, Abbasi and Hadian Rasanani [47] analysed lumbar proprioception using angular repositioning errors following the application of Kinesio Taping in subjects with chronic lower back pain, but found no significant improvements. Another methodologically important aspect is that the instruments are not equivalent in terms of the neurosensory subsystems they involve or stimulate. Whilst strength tests (such as those involving the reproduction of 50% of maximum voluntary force) primarily stimulate muscle spindles and Golgi tendon organs, passive joint position tests tend to involve joint and cutaneous receptors [43]. Many assessments do not adequately control for external sensory or motor variables, which calls into question the specificity of tests labelled as proprioceptive [26]. This distinction is important when selecting the appropriate instrument according to the clinical or research objective. In terms of population, most tests have been developed and validated in young healthy adults. This raises doubts about their general applicability in populations with neurological disorders, older adults, or patients with chronic pain conditions, where sensorimotor mechanisms are impaired [46,47]. Similarly, there are a lack of longitudinal studies evaluating proprioceptive change in response to specific therapeutic interventions.

Ultimately, the main limitation in the assessment of proprioception lies in the fact that there is no single instrument capable of comprehensively capturing the multisensory and sensorimotor complexity that defines it. Current tools isolate specific submodalities or employ simplified tasks, which compromises their ecological validity and limits their transferability to clinical practice. Until integrative models that take the functional context into account are developed, clinical decisions must be based on the critical combination of multiple tests. Although this integration facilitates a broader characterisation of the phenomenon, it does not eliminate the limitations inherent in each instrument, nor does it guarantee a complete understanding of proprioceptive behaviour under real-world conditions.

**Figure 2:**
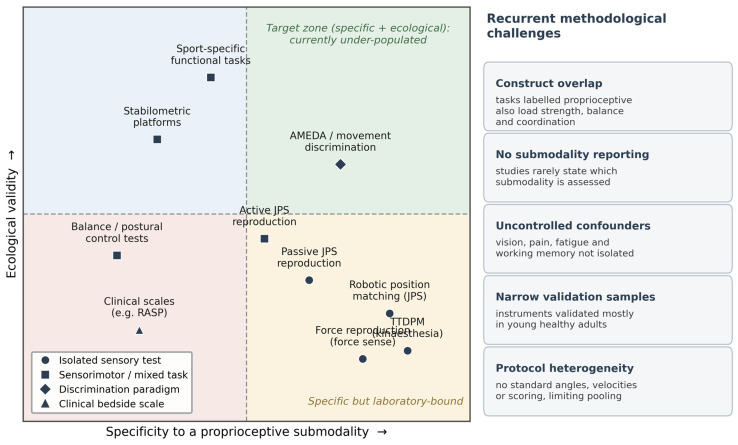
Limitations of current proprioceptive assessment methods: ecological validity, specificity and methodological challenges. The grey dashed lines divide the plane into quadrants; they are reference axes rather than arrows. The horizontal axis represents ecological validity and the vertical axis submodality specificity, both increasing towards the upper right.

To summarise the main limitations associated with current proprioceptive assessment methods, including issues related to ecological validity, specificity and methodological consistency, a conceptual overview is presented in Figure 2.

The figure summarises the main limitations associated with commonly used proprioceptive assessment tools. Current methods vary widely in their ecological validity and specificity to proprioceptive submodalities, with many assessments conducted under controlled laboratory conditions that do not reflect real-world motor demands. Additionally, the lack of standardisation and the frequent overlap with general sensorimotor constructs limit the ability to isolate and accurately measure true proprioceptive function.

To make these limitations directly comparable across instruments, and in response to the heterogeneity described above, Table 1 contrasts the main assessment methods in terms of the submodality each one actually targets, the psychometric performance reported for it, whether the evidence base employed control groups, and its ecological validity. Two patterns emerge. First, precision and ecological validity are inversely related across almost the entire set of instruments: the methods that isolate a submodality most cleanly are the least representative of the conditions in which proprioception is actually used. Second, the instruments most frequently used to justify claims about proprioceptive training, namely balance and posturographic indices, are precisely those that isolate no submodality at all, which makes the attribution of any observed change to a proprioceptive mechanism logically unsupported.

## 7. Proprioception and Sport

Proprioception, and the clear lack of consensus regarding its definition, could be understood from a sporting perspective as the body’s ability to perceive its position and movement in space; it is a neural process by which the body senses a series of environmental stimuli and integrates them to adapt movements in response to what has been perceived [51,52], thus playing a crucial role in elite sporting performance, as proprioception (ankle movement discrimination tests) is associated with the level of sporting performance achieved [48,53] to the extent that athletes have developed this ability to a greater or lesser degree, it will be reflected in higher-quality technical and tactical actions, and similarly, they tend to suffer fewer injuries. However, despite its importance, there are notable differences in the explanation, understanding and application of this concept to sport, leading to confusion amongst coaches and athletes regarding how to develop it. This inconsistency not only affects how proprioception is trained, but also influences injury prevention and performance optimisation.

In this chapter, we will explore the multiple facets and contradictions found in proprioception in sport, analysing how differences in the interpretation and practice of this phenomenon can impact both athlete development and competitive results.

As mentioned previously, in sport, one of the main tools used is proprioception as an alternative to reduce or prevent injuries; terms such as ‘prophylactic proprioception’ have even been established, the aim of which is to carry out interventions to prevent injuries [52]. However, in epidemiology, from a sporting perspective, participants who undergo interventions for injury prevention can be categorised into four groups. ‘Participants would be categorised as “Doomed” if they would be injured with or without the intervention, “Immune” if they would remain uninjured with or without the intervention, “Preventive” if they would remain uninjured with the intervention but would be injured without it, and “Causal” if they would be injured with the exercise intervention but would remain uninjured without it’ [54]. Consequently, there is a very noticeable bias in most studies that establish this hypothesis of ‘proprioception for the prevention of sports injuries’, as it is not established whether there is an acute causal effect for each participant, determining whether the training is beneficial or harmful (preventive or causal).

Now, in the quest to work on and strengthen proprioception, a series of training programmes are often established, which lack an established framework or method to organise proprioceptive training in a quantifiable manner and develop it in the best possible way. The basis is established by exercises that challenge stability, improve neuromuscular function, activate stabilising muscles and require constant postural adjustment, particularly in joints such as the ankle, knee and hip [52,55]. However, there is no theoretical basis from which to start a series of progressions or regressions that would allow a clear path to be mapped out. Strength exercises such as squats, lunges and even unilateral balance are commonly used. The question is, at what point does a strength exercise such as the squat neglect the proprioceptive component, given that, in essence, for its correct execution, the agonist, synergist and antagonist muscles must perform their function properly (stability and balance). The same applies to exercises such as scissors or lunges, the push jerk, the snatch in weightlifting, etc., which involve the entire locomotor system neuromuscularly and which must be primed to receive signals in order to respond to the demands of movement and load mobilisation during the movement. So, is strength training, in turn, proprioceptive training? Or simply, must one work on unstable surfaces as a fundamental rule for it to be considered proprioceptive training?

Another option that has emerged to enhance proprioception and thereby improve sporting performance involves the use of external aids such as kinesiotaping, compression socks, latex splints, rigid tape, etc. These types of passive interventions [49] have emerged with the aim of achieving positive effects (often without being statistically significant) on proprioception, balance, joint stability, ‘amplification of sensory information’, optimisation of movement and reduction in load on joint structures, as well as other aspects that make up athletes’ proprioception; even improvements in vertical jump (CMJ) have been observed when using kinesiology tape or patellar straps [50,56,57,58,59]. Others directly highlight the benefits of neoprene shorts or compression stockings and how they influence leg proprioception, asserting that their use provides a benefit in the proprioceptive control of the ankle joint after a certain distance and in the precision of leg balance in subjects with low neuromuscular control capacity, whereas those with developed capacity actually experience a reduction in control [50,51,60].

Further research continues to show that the guidelines for the use and application of kinesiotape, rigid tapes, splints and all such passive aids are inconsistent; the methodological quality is not reliable enough to support the evidence attributed to the results obtained [61,62,63,64]. Perhaps this is because they provide a momentary stabilising effect that gives the patient or athlete, in this case, a perception of stability [65,66].

All these types of external aids are intended to act as proprioceptive enhancers to improve sporting performance. Bandages, compression socks or shorts appear to provide excellent and immediate results, which should be considered by coaches when aiming to improve proprioception. But what do they actually improve? The stability they provide—to what extent can this be considered a placebo designed to give the athlete momentary confidence without directly addressing the problem? What happens, and what risk does the athlete face when they decide to stop using the support? If positive results are seen in improved stability, neuromuscular activation and the perception of movement in terms of timing, space, force and speed of execution, how necessary would it actually be to seek benefits from proprioception training if the solution lies in these types of aids? To put forward this hypothesis is to bypass the step of generating a series of relevant neurological and physiological stimuli that would actually adapt the body to the demands of the sport; it completely overlooks basic processes such as the adaptation process [67]. For example, small but statistically significant benefits are found in athletes whose proprioception is not highly developed in the joint presenting the problem; conversely, those athletes who possessed a solid foundation in terms of proprioceptive development performed worse in the tests [57,68]. What about those who do possess good proprioceptive ability, such as high-performance athletes? Is sports taping then a temporary stepping stone on the path for all athletes whilst optimal proprioceptive development is consolidated? Concerns arise, such as in the case of young athletes aiming for high performance, whose stage of development (puberty, for example) requires an adaptation in proprioception due to their constant physical changes. Should they be the first to use this type of aid, given that it is established to aid proprioception?

**Figure 3:**
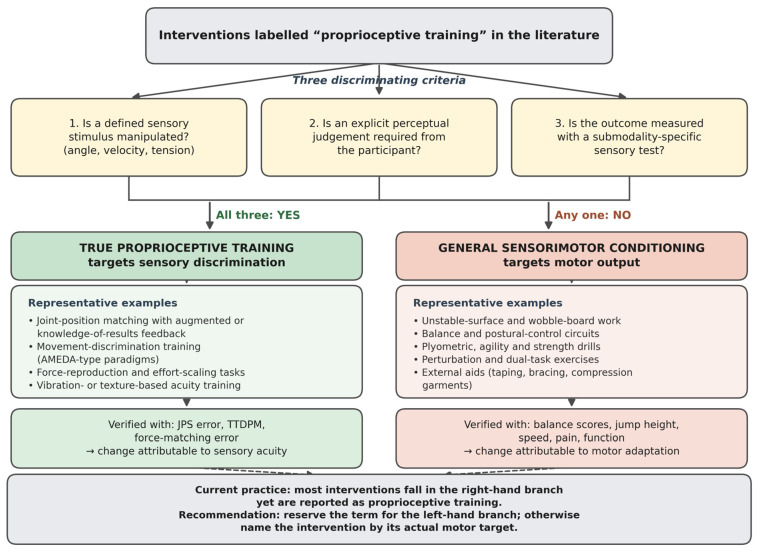
Taxonomy of ‘proprioceptive training”. Solid arrows indicate the classification pathway from the interventions labelled ‘proprioceptive training’ through the discriminating criteria to the two resulting branches; the dashed arrows indicate that both branches feed the summary statement shown at the foot of the figure.

Despite the widespread use of the term ‘proprioceptive training’, current practice often lacks conceptual clarity, with a large proportion of interventions targeting general motor capacities rather than specific proprioceptive processes. This mismatch between terminology and underlying mechanisms is illustrated in Figure 3.

## 8. Results

Following the search described in Section 2.5, the studies on proprioception in rehabilitation and readaptation listed in Table 2 were identified; the corresponding evidence on injury prevention and sports performance is presented in Table 3.

### 8.1. Proprioception and Rehabilitation

#### 8.1.1. Rehabilitation and Readaptation

Analysis of the included studies reveals marked heterogeneity in programmes labelled as ‘proprioceptive training’, both in their design and in their components, duration, frequency and intensity. This variability prevents the establishment of a consensus or clear standardisation that would allow proprioceptive training to be recognised as a specific intervention modality. On the contrary, the protocols reviewed comprise a wide range of sensorimotor strategies that share the general objective of improving joint stability and neuromuscular control, but differ substantially in their theoretical basis, methodological structure and evaluation criteria.

The rehabilitation programmes analysed ranged from two to six sessions per week, with durations of 2 to 24 weeks and sessions lasting 10 to 60 min (which truly hinders the reproducibility of the interventions). Progression is based on the complexity of the stimulus rather than on external load, moving from stable conditions with visual control towards tasks involving disturbances, single-leg support, sensory deprivation or dual tasks. In many cases, the exercises are combined with strength, coordination and balance training, or with complementary modalities such as vibration platforms, visual feedback systems, PNF or interactive environments.

However, the lack of standardised protocols, valid measurements and clear operational definitions prevents us from affirming the existence of ‘proprioceptive training’ in the strict sense. What is currently termed as such corresponds rather to multimodal neuromuscular interventions that indirectly stimulate the sensory pathways involved in proprioception. This conceptual ambiguity limits the interpretation of results, as the observed benefits (improvements in balance, stability or functional performance) could stem from global motor adaptations rather than sensory changes per se.

Furthermore, it should be clarified that the use of unstable surfaces is neither the only nor the best way to improve proprioception. Whilst they may increase sensory integration demands, their use without a clear purpose or appropriate dosage may induce motor compensations rather than optimising proprioceptive sensitivity. Therefore, the evidence suggests that efficacy depends more on planned and contextualised sensorimotor progression than on the type of surface used.

Overall, the programmes reviewed promote functional rehabilitation and a safe return to sporting activity, but their results must be interpreted with caution given the lack of methodological consistency and specific measurements. Thus, it can be stated that so-called proprioceptive training does not exist as a homogeneous entity, but rather as a family of sensorimotor strategies whose efficacy depends on the population, the stimulus and the coherence between mechanism and outcome, rather than on a universal methodological model.

This lack of conceptual and terminological consistency not only limits the interpretation of results but also runs counter to the basic principles of evidence-based medicine, which require rehabilitation programmes to be built on verifiable criteria, defined phases with specific objectives, progression criteria, valid measurement tools, and dosing strategies that ensure load control and adaptive tissue response. However, in the context of so-called ‘proprioceptive training’, such principles are rarely met. Most protocols lack defined entry and exit criteria for each phase, use indirect or non-specific measures of proprioception, and feature poorly standardised progression, making it difficult to establish with certainty the presence of temporary and objective improvement in our patient. This methodological shortcoming contradicts the fundamentals of evidence-based rehabilitation and demonstrates the need for a conceptual redefinition of the term.

Beyond the absence of methodological criteria, assuming that a single “proprioceptive training” approach can be applied indiscriminately to different conditions constitutes an oversimplification. The adaptive response of a reconstructed anterior cruciate ligament differs significantly from that of a patient with chronic low back pain or an individual with sequelae of a cerebrovascular event. Each of these clinical conditions involves tissues with different biological healing times and load tolerance during rehabilitation [69], different predominant types of mechanoreceptors and degrees of heterogeneous neural plasticity [70].

We might question whether, in patients with meniscal injuries where joint mechanoreceptors are compromised [71], in those with diabetic polyneuropathy where afferent input from muscle spindles and cutaneous receptors is altered [72] or in subjects with central neurological damage [73], ‘proprioceptive training’ programmes would yield the same result, despite the differences in the nature and origin of their deficits.

**Table 2:** Studies focusing on rehabilitation and their load parameters. N/A and ‘Not applicable’ indicate that the parameter was either genuinely not applicable to the protocol or, more often, not reported in the source study; the two could not be distinguished from the published reports in every case. The frequent absence of reported load parameters is itself one of the findings discussed in Section 8.1.3.

Reference	Frequency	Intensity	Time	Type	Volume	Rest
Duray, M., Simşek, S., Altuğ, F., & Cavlak, U. (2018) [74]	5 sessions per week × 3 weeks	N/A	10 min	GDRE gaze direction recognition exercise	Not applicable	Not applicable
Sipko, T., Glibowski, E., & Kuczyński, M. (2021) [75]	Not applicable	Not applicable	30 min	PNF	5–8 repetitions per exercise	N/A
Pistone, E. M., Laudani, L., Camillieri, G., Di Cagno, A., Tomassi, G., Macaluso, A., & Giombini, A. (2016) [76]	3 sessions per week × 4 weeks	Median of 35 Hz, amplitude 2 mm	1 min	Whole-body vibration protocol at optimal frequency (WBV-of).	3–10 sets depending on the week	1 min
Baltaci, G., Harput, G., Haksever, B., Ulusoy, B., & Ozer, H. (2013) [77]	3 sessions per week for 12 weeks	N/A	1 h	Interactive games with Nintendo Wii Bowling, Skiing (Wii Sports), Boxing, Football, Balance Board (Sports Pro Series)	15 min per game	N/A
Moezy, A., Olyaei, G., Hadian, M., Razi, M., & Faghihzadeh, S. (2008) [78]	3 sessions per week × 4 weeks	30 Hz to 50 Hz	4–16 min	The WBVT group exercised on a vibration platform a: semi-flexed standing b: single-leg stance with knees slightly bent c: mini squat. d: single-leg mini squat. e: deep squat. f: single-leg deep squat. g: wide squat. h: lunge. i: tiptoes.	30–60 s per set. 8–19 sets depending on the week	30 to 60 s
Akbari, A., Ghiasi, F., Mir, M., & Hosseinifar, M. (2015) [79]	6 days a week for 2 weeks	N/A	30 min	Single-leg balance with eyes open and closed. Anterior, lateral, and posterior step-ups with the operated and non-operated leg.	Not applicable	Not applicable
da Silva Boitrago, M. V., de Mello, N. N., Barin, F. R., Júnior, P. L., de Souza Borges, J. H., & Oliveira, M. (2021) [80]	3 sessions per week for 6 weeks	Standardized at 70% of 1RM; intensity increased every 2 weeks	60 min (10 min for proprioception)	2 Proprioceptive exercises (Weeks 1–6) single-leg balance on a stable surface with knee flexion (30°) single-leg balance on an unstable surface with knee flexion (30°)	3 sets × 15 repetitions of each exercise	30 s
Zarei, H., Norasteh, A. A., Lieberman, L. J., Ertel, M. W., & Brian, A. (2023) [81]	3 sessions per week × 8 weeks	From stable to unstable surfaces	60 min	Parallel bars swiss balls incline board wobble board boss balls eye masks	Time: 2 sets of 15 s Repetitions: 2 sets of 10–15 reps	1 min
Cooper, R. L., Taylor, N. F., & Feller, J. A. (2005) [82]	2 sessions per week for 6 weeks	From stable to unstable surfaces	60 min per session	Easy bicycle (no hands) to moderate bicycle (no hands); double-leg standing on mini-trampoline while touching a ball to single-leg standing on mini-trampoline while touching a ball; double-leg standing on wobble board to double-leg standing on wobble board with eyes closed; double bridge on fitball to single-leg bridge on fitball; wall squats on a fitball to single-leg squats with back leg on a fitball; walking on a balance beam to walking with various equipment (mini trampoline, Dura Disc, wobble board). At home: easy walking 20–30 min to moderate-to-vigorous walking 40 min; standing on both feet on a mat while throwing a ball to standing on one foot on a mat while throwing a ball; standing on both feet on two rolled-up towels to standing on one foot on a rolled-up towel with and without eyes closed; double bridge on a fitball to single-leg bridge on a fitball; wall squats on a fitball to single-leg squats with the back leg on a fitball; heel-toe walking with eyes closed without changing feet.	Not Applicable	Not Applicable
Vathrakokilis, K. & Malliou, Paraskevi & Gioftsidou, Asimenia & Beneka, Anastasia & Godolias, G. (2008) [83]	2 times per week for 6 weeks	Increasing the balance demands of the exercise by decreasing the base of support	20 min	Static single-leg 2 min on a hemispherical cylinder (restricted anterior-posterior movement). Dynamic single-leg balance for 2 min on a hemicylindrical board (restricted anterior-posterior movement). Static single-leg 2 min on a hemicylindrical board (medial-lateral movement restricted). Dynamic single-leg stance for 2 min on a hemispherical board (medial-lateral movement restricted). Static single-leg balance for 2 min on a hemispherical board (free movement in both directions).	Not applicable	Not applicable
Bitterli, R., Sieben, J. M., Hartmann, M., & de Bruin, E. D. (2011) [84]	Daily for 2–6 weeks	Not applicable	Not applicable	The program included six exercises: lying down, contracting the legs and glutes; moving the operated leg outward and returning it; sliding the foot forward and backward with the knees bent; and lifting the glutes to form a bridge. Standing, bending and extending the hips and knees with legs apart, and finally leaning on the healthy leg while moving the other to the side and returning.	2 sets x 10 repetitions per exercise [6]	Not applicable
Gstoettner, M., Raschner, C., Dirnberger, E., Leimser, H., & Krismer, M. (2011) [85]	Once a week for 6 weeks	Start on stable surfaces with eyes open, progress to unstable surfaces (Airex mat/pad) and with eyes closed if possible, gradually increasing speed and range in exercise 2.	45 min	Balance and proprioception training: Barefoot on: Hard floor airex mat (1.5 cm) airex pad (6 cm) support is allowed if necessary. Start with eyes open and progress to eyes closed if possible. Exercises: (1) slide the foot forward and backward without lifting it, alternating legs; (2) take short steps forward and backward, raising the knee to 90°, 10–15 repetitions per side; (3) balance on one leg with the knee at 90° for at least 10 s, alternating; and (4) take a lateral step of ~30 cm, hold for 10 s before returning and switching sides.	3 sets, 10 to 15 repetitions (isometric 10 s),	Depending on the patient
Huber, E. O., Roos, E. M., Meichtry, A., de Bie, R. A., & Bischoff-Ferrari, H. A. (2015) [86]	2–3 weeks, 2 sessions per day	3 difficulty levels, varying the number, direction, and speed of the movement, changing the surface; progression was made when the exercise achieved 3 sets of 15 repetitions with good neuromuscular control and proper execution	Not applicable	Begins with a 10 min aerobic warm-up on a stationary bike, followed by a circuit of 4 exercises, ending with a 10 min cool-down. Focus: postural stability, functional alignment, upper and lower body strengthening, and functional exercises. Neuromuscular exercises in closed kinetic chains, with an emphasis on postural control, functional strength, and stability	2–3 sets, 10–15 repetitions	Between exercises and circuits
Jogi, P., Overend, T. J., Spaulding, S. J., Zecevic, A., & Kramer, J. F. (2015) [87]	1–2 times per week for 5 weeks	Light to moderate, progressive based on functional tolerance and therapist supervision	Not applicable	Hip: assisted knee flexion and extension, isometric quadriceps, abduction, hip extension and flexion while standing with support. Knee: assisted knee flexion and extension, straight leg raise, flexion by sliding the foot on the bed and floor, extension while seated. Balance exercises for both: Trunk rotation while standing without support (clockwise and counterclockwise). Unassisted walking lunges, alternating legs forward. Shift weight to one side while standing, repeat on the other side.	3 sets of 10 repetitions	N/A
Liao, C. D., Liou, T. H., Huang, Y. Y., & Huang, Y. C. (2013) [88]	3–5 times per week for 8 weeks	The intensity is moderate and adapted to the patient’s capacity, focused on gradually improving postural control and proprioception without causing excessive fatigue	90 min	Traditional training + Side stepping, Braiding activities, Tandem walk, Cross-over steps × 5 min, improves dynamic balance and gait, weeks 1–5, Shuttle walking: 5 min, strengthens gait, weeks 3–5,Multiple changes in direction, Foam activity: 5–10 min, improves posture and proprioception, weeks 4–6, BAPS/Tilt board activity, Balance beam walk 10 min, strengthens proprioception and balance, weeks 6–8.	5–10 min depending on the exercise	Not applicable
Piva, S. R., Gil, A. B., Almeida, G. J., DiGioia, A. M., 3rd, Levison, T. J., & Fitzgerald, G. K. (2010) [89]	2 sessions per week for 6 weeks	Progression is individualized depending on the patient’s ability and stability when performing the exercises	Not applicable	Traditional training + 1. Side stepping 2. Braiding activities 3. Tandem walk 4. Cross-over steps 5. Shuttle walking 6. Multiple changes in direction 7. Foam activity 8. Tilt board activity 9. Roller board and platform perturbations	2 times per direction or 30 s in duration	Not applicable
Villadsen, A., Overgaard, S., Holsgaard-Larsen, A., Christensen, R., & Roos, E. M. (2014) [90]	2 sessions × 8 weeks preoperatively	Progression based on movement quality and pain (VAS < 5),	1 h	NEMEX-TJR: 10 min of aerobic warm-up on a stationary bike. Exercise circuit focused on 4 main areas: Core stability/postural control. Postural alignment. Lower-body muscle strength. Functional exercises.	2–3 sets, 10–15 repetitions	Between exercises and circuits
Ordahan, B., Küçükşen, S., Tuncay, İ., Salli, A., & Uǧurlu, H. (2015) [91]	24 weeks	N/A	Not applicable	0–2 weeks: Edema control (cryotherapy, elevation), ankle mobility, straight-leg raises in 4 directions, isometric exercises for the hips, quadriceps, hamstrings, and adductors. 3–5 weeks: Joint mobility (wall/heel glide), bicycle, initiation of proprioception (single-leg balance, heel/toe walking, eyes closed, backward walking), and weight-bearing strengthening. Weeks 6–8: Removal of splint and crutches, plus proprioception (drawing a figure-of-eight with feet, 15 cm jump, unstable board). 9–12 weeks: Functional walking (sideways, backward, sidelaps), resistance training, walking in water. 13–19 weeks: Advanced proprioception (trampoline, unstable board), single-leg and double-leg jumps, plyometric exercises. 20–24 weeks: Progressive return to sports activities.	Not applicable	Not applicable
Risberg, M. A., Holm, I., Myklebust, G., & Engebretsen, L. (2007) [92]	6 months × 2 sessions per week	Not applicable	Not applicable	The Neuromuscular Training (NT) Group, based on 6 progressive exercises focused on postural control, dynamic balance, and coordination, using unstable platforms such as BOSU and integrating sensorimotor stimuli (dual tasks and perturbations). The goal was to improve anticipatory activation and motor response, particularly in stabilizing muscles such as the peroneals, with personalized progression based on functional level. The Strength Training (ST) Group used elastic bands to strengthen ankle muscles (dorsiflexors, plantarflexors, inverters, and everters), including simple static proprioception exercises. Progression focused on increasing resistance, repetitions, and sets, without incorporating instability or complex stimuli.	Not Applicable	Not Applicable
Kaya, D., Guney-Deniz, H., Sayaca, C., Calik, M., & Doral, M. N. (2019) [93]	Follow-up between 1.5 and 12 months: 2 to 7 times per week	N/A	15 to 60 min per session	Proprioceptive Training Group (Experimental) duration of intervention (range in studies): From the 3rd week post-surgery to the 4th year. Follow-up between 1.5 and 12 months. Frequency and intensity: Main exercises: Static and dynamic balance training. Squats, lunges, exercises on unstable surfaces. Balance beam walks, jumps, plyometric exercises. Agility drills. Sensory stimulation (sometimes combined with PNF). Neurocognitive and functional rehabilitation focused on sports tasks. In some cases: vibration and biofeedback training.	Not applicable	Not applicable
Zheng, Q. Y., Sun, J. N., Wang, R. S., Ma, Y. R., & Chen, P. (2025) [94]	4 times a week for 12 weeks post-operative	Not applicable	15 min daily	Traditional training + 0–2 weeks of traditional exercise only, proprioception: weeks 3–5 single-leg standing (30 s) balance reach leg balance reach arm bilateral squat walking backward with eyes open (≥15 min daily) 6–12 weeks: Single-leg standing ≥30 s single-leg pelvic bridge on the surgical side (≥30 s) balance reach leg balance reach arm single-leg squat slide skip walking backward (≥15 min daily) balance board exercises ≥15 min daily	3 sets of 15 repetitions, balance and walk for 15 min	Not applicable
Bąkowski, P., Ciemniewska-Gorzela, K., Bąkowska-Żywicka, K., Stołowski, Ł., & Piontek, T. (2021) [95]	24 weeks	Not applicable	Not applicable	Five progressive phases. Starting in Phase I, isometric exercises and early joint mobility exercises were performed. In Phase II, active exercises, hip exercises, and core stability exercises were incorporated, along with the first proprioceptive exercises in Group A. Phase III included standing exercises on force plates and unstable surfaces, as well as squats up to 90°. In Phase IV, external load, closed-chain exercises, stationary bike with resistance, and functional exercises on stable and unstable surfaces were added. Finally, Phase V introduced bipedal and unipedal jumps and plyometric exercises to develop power and dynamics.	N/A	Not Applicable
Dragicevic, Dragana & Erceg-Rukavina, Tatjana & Nikolić, Siniša. (2021) [96]	5 times per week	N/A	10 min	Biodex 4 Pro: Recognize and maintain flexion angles (15°, 30°, 45°): Seated position with torso, thigh, and leg secured with straps. Range of motion: 0° (extension) to 90° (flexion). Exercises with eyes open and closed In the gym: on unstable surfaces exercises on balance balls, balance boards, and wobble boards without a dynamometer.	Not applicable	Not applicable
Rexha, E. (2024) [97]	12 weeks × 5 times a week	Not applicable	Not applicable	Reeman Table, Bosu, resistance bands, balance boards, etc. The exercises are conducted in a gradual and controlled manner	8–10 repetitions × 3 sets	Not applicable
Lim, J. M., Cho, J. J., Kim, T. Y., & Yoon, B. C. (2019) [98]	6 months	Progressive	1 h	Education: Q-setting (co-activation), seated hamstring stretch, full knee extension phase 1: Q-setting, 60° mini-squat, SLR (front, side, back), hamstring stretch, ROM exercise up to 90°, seated knee flexion phase 2: Stationary bike 15 min, SLR with elastic bands, squats up to 60°, knee flexion/extension with band, stretching, ROM up to 120°, single-leg balance phase 3: Stationary bike 15 min, 60° squats, knee flexion/extension with band, 60° lunges, stretching, single-leg balance with balance board phase 4: Stationary bike 15 min, 90° squats, full-range knee flexion/extension with resistance band, 90° lunges, stretching, single-leg balance with balance board, treadmill jogging up to 20 min	Not applicable	Not applicable

To claim that a single type of training with “proprioceptive exercises” is equally effective for all tissues and pathologies ignores the multimodal and hierarchical nature of the somatosensory system and its complexity. If the afferent and efferent mechanisms involved in cortical and peripheral reorganisation differ between musculoskeletal and neurological injuries, rehabilitation protocols should, consequently, be directed towards proprioceptive submodalities (sense of position, sense of movement and sensation of effort or force) [43], which would allow for the development of a more logical approach in line with the physiology of the proprioceptive system, opening up a range of therapeutic possibilities with better-defined strategies and more specific rehabilitation protocols.

**Figure 4:**
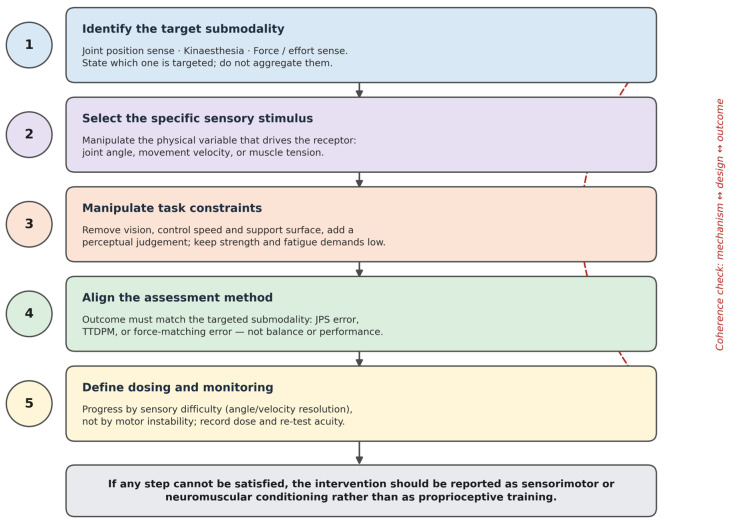
Proposed framework for proprioceptive training. Solid arrows indicate the sequential order of the five steps; the red dashed line indicates the coherence check that must hold between mechanism, design and outcome across the whole sequence.

Given the lack of conceptual clarity and methodological consistency identified across the literature, a structured framework is proposed to guide the design, implementation, and evaluation of proprioceptive training interventions (Figure 4).

The figure presents a stepwise framework for the development of proprioceptive training programs, beginning with the identification of the target submodality (joint position sense, kinaesthesia, or force perception), followed by the selection of specific sensory stimuli, manipulation of task constraints, alignment of assessment methods, and integration of dosing and monitoring strategies. This approach aims to ensure coherence between the underlying neurophysiological mechanisms, the intervention design, and the outcome measures, facilitating the differentiation between true proprioceptive adaptations and general motor improvements.

#### 8.1.2. Injury Prevention and Sports Performance

In the field of injury prevention and sports performance, the evidence continues to show a trend towards heterogeneity and a lack of consensus. Programmes vary in duration (4 to 16 weeks), frequency (2 to 5 sessions per week) and structure, with no uniform criteria regarding dosage, type of task or control variables. This methodological diversity confirms that there is no single, replicable or standardised proprioceptive training programme, but rather multiple approaches that combine balance, coordination, strength and agility exercises with cognitive or sport-specific tasks.

Whilst several studies report reductions in the incidence of injuries (particularly ankle and knee sprains) and improvements in postural control and dynamic stability, the results regarding pure performance (speed, jumping or maximum strength) are inconsistent. This suggests that the benefits of so-called “proprioceptive training” are expressed primarily as improvements in motor efficiency and functional stability, rather than as direct increases in physical performance.

Similarly, the repeated use of unstable surfaces or balance exercises as a central focus reflects a reductionist interpretation of the concept of proprioception. Current findings make it clear that proprioceptive stimulation can be achieved through multiple means—external perturbations, dynamic tasks, sensory feedback, sport-specific movements—without relying exclusively on instability. Therefore, the most appropriate approach appears to be the integration of the proprioceptive component within multimodal programmes, encompassing strength, motor control, coordination and sensory re-education.

In the context of football, for example, a player running towards the ball who is intercepted by an opponent suddenly changing direction must immediately activate anticipatory control and reflex mechanisms to reorganise their motor pattern within a matter of milliseconds. This rapid adjustment involves detecting visual and vestibular changes, integrating information from the somatosensory system, and generating a motor response capable of maintaining dynamic stability [70,99]. If the somatosensory system, based on the interactions of afferent and proprioceptive inputs, fails to effectively anticipate or compensate for this change due to fatigue, proprioceptive deficit or reduced nerve transmission of the afferent impulse, the risk of loss of stability increases and the chaos that precedes many musculoskeletal injuries [100,101].

This example highlights the essence of motor control and its relationship with stability in the sporting context. But what do we mean by stability? From a mechanical perspective, a system is considered stable when, following a disturbance, it manages to recover its initial state of equilibrium. This process requires the sensorimotor system to generate compensatory forces to counteract external disturbances. During the execution of a movement, the structure undergoes a transient imbalance, known as dynamic equilibrium, in which stability depends on the body’s ability to continuously re-establish postural and joint control [99].

Based on the above, the literature reveals a clear conceptual inconsistency between static balance, dynamic balance and the term ‘stability’. Although these concepts are related, they are not equivalent, yet many ‘preventive’ or ‘proprioceptive training’ programmes use them as if they were. Most are limited to balance exercises or tasks on unstable surfaces, vibrating platforms and other resources which, whilst useful in certain contexts, do not encompass the complexity of the somatosensory system or the actual physiology of proprioception.

This confusion has led to reductionist interpretations of the categories of motor control, resulting in interventions that are not always consistent with the neurophysiological mechanisms that actually underpin joint stability and performance. So, should prevention and performance focus solely on improving balance or stability, whether static or dynamic? Or, conversely, should we prepare the athlete to perform in changing environments and under conditions of fatigue, optimising the nervous system’s ability to anticipate, interpret and respond to the disturbances inherent in sport?

We should not take a simplistic view of just how amazing our somatosensory system is; the concept of functional joint stability involves far more structures than simply joints that are congruent with one another and provide mechanical stability. We now know that there is much more to it, and that the role of tendons, muscles, cartilage and other structures in stability is far more significant than we previously thought [102].

This neurofunctional approach, based on the principles of feedforward control (a system that predicts and prepares) and feedback control (reacts and corrects), redefines the role of the misnamed ‘proprioceptive training’ and paves the way for a new approach to training, orienting it towards the development of adaptive sensorimotor control strategies [70,103], tailored to the actual physical demands of the sporting movement.

From a neurophysiological perspective, functional joint stability depends on the interaction between two control mechanisms acting simultaneously: the anticipatory (feedforward) and the feedback (feedback) mechanisms. The first prepares the system through anticipatory neuromuscular contraction, increasing muscle stiffness and sensitivity to disturbances or unexpected impacts, thereby enhancing joint stability before the mechanical stimulus occurs. The second mechanism, feedback control, is activated immediately following a disturbance through the stimulation of mechanoreceptors and spinal reflexes, facilitating rapid correction of movement and the restoration of joint balance [70,104].

Together, both systems form the basis of dynamic stability and highlight the brain’s essential role in motor control [105], aspects that should be considered when designing prevention and performance programmes [106]. Training balance or strength alone, without integrating anticipatory preparation and reflexive response, limits the actual adaptation of the sensorimotor system and may leave the athlete vulnerable to unexpected disturbances. Much remains to be discovered, but this model brings us closer to a deeper understanding of the neurophysiology of somatosensory control and offers valuable guidance for mitigating sports injuries and optimising human performance.

#### 8.1.3. Integrative Methodological Note

This review highlights that there is no such thing as ‘proprioceptive training’ in the strict or standardised sense. Rather, the term encompasses a constellation of heterogeneous interventions, with no consensus on their definition, structure, dosage, or valid measurement of outcomes. The lack of clear protocols and specific indicators of sensory change prevents the delineation of their mechanisms and actual effects. Therefore, the understanding of proprioceptive training should shift from a narrow technical notion towards a broad, contextual sensorimotor approach, which recognises the dynamic interaction between perception, movement and motor learning as the core of the rehabilitation and prevention process.

**Table 3:** Studies focusing on training and its load parameters. N/A and ‘Not applicable’ indicate that the parameter was either genuinely not applicable to the protocol or, more often, not reported in the source study; the two could not be distinguished from the published reports in every case. The frequent absence of reported load parameters is itself one of the findings discussed in Section 8.1.3.

Reference	Frequency	Intensity	Time	Type	Volume	Rest
Antohea, B. A., Rață, M., Rață, B. C., & Rață, G. (2023) [107]	15 weeks	Intensity is based on progression:—Start: Static, two-legged exercises with eyes closed6.—Progression: Dynamic, one-legged exercises with eyes closed6.—Use of unstable surfaces (such as balance boards and discs)6.	15/20 min	Progressive exercises: static, two-legged exercises with eyes closed, and ending with dynamic exercises with eyes closed. Exercises performed: dynamic and static balance exercises, progressing from one-legged to two-legged on unstable surfaces, using: balance board, inflatable balance disc, gym bench, balance brick, modular/agility ladder for speed training, rhythmic gymnastics circle, and plastic hurdles	10 to 15 repetitions	Not applicable
Ghaderi, M., Letafatkar, A., Almonroeder, T. G., & Keyhani, S. (2020) [108]	2 to 3 sessions per week for 8 weeks	Moderate to high and progressive	30 to 45 min	Two-legged and one-legged squats, lunges, jumps, balance on an unstable surface, horizontal bounds, long jumps	3 to 5 sets, 6–12 repetitions or 30 s balance	30–60 s between sets
Gidu, D. V., Badau, D., Stoica, M., Aron, A., Focan, G., Monea, D., Stoica, A. M., & Calota, N. D. (2022) [109]	4 times per week for 8 weeks	Without ball on BOSU a with ball	30 min	Subprogram 1—Without ball: Balance and strength exercises on the BOSU, such as squats (two-legged and one-legged), leg swings, and jumps (forward, side, and lunge-style). Subprogram 2—With ball: Exercises combining proprioception with soccer skills, such as kicking and shooting on the BOSU, jumping between BOSUs with a ball, and ball control with resistance. THERE WERE 12 EXERCISES ON A BOSU	4 sets of 10 repetitions	30 s between each set
Souglis, A. G., Travlos, A. K., & Andronikos, G. (2023) [110]	16 weeks × 5 sessions per week	N/A	20 min	Ladder drills, drills with hoops, and balance exercises on BOSU, TOGU, inflated balance discs, and mini trampoline. 15-min warm-up: • Agility ladder (8 types: 1–2 foot sprints, hops, slalom, diagonal, lateral, single-leg, straddle). • Hoop drills: 2 different routes, followed by a 10-m fast dribble and a shot on goal. • Balance exercises on BOSU: one-legged balance, foot and head passes, pull-ups, shots. • Same sequence on TOGU, inflatable discs, and trampoline. • Training divided into 6 field stations with 2 stations each (12 stations total: 2 ladders, 2 hoops, 2 BOSU balls, 2 TOGU balls, 2 discs, 2 trampolines).	30 s	N/A
Harry-Leite, P., Paquete, M., Teixeira, J., Santos, M., Sousa, J., Fraiz-Brea, J. A., & Ribeiro, F. (2022) [111]	N/A	N/A	10 min	1. BOSU Squat 2. Two-legged balance on the BOSU 3. Throwing and catching the ball while in a two-legged stance on the BOSU 4. One-legged balance on the BOSU 5. Throwing and catching the ball while standing on one leg on the BOSU 6. Jump onto the BOSU in a two-legged stance 7. Jumping onto the BOSU in a one-legged stance 8. Jump onto the BOSU in a two-legged stance with a 90° trunk rotation 9. Two-legged balance on the BOSU with eyes closed 10. One-legged balance on the BOSU with eyes closed	10/60 s	N/A
Antohe, B. A., & Panaet, E. A. (2024) [112]	15 weeks × 3 sessions per week	Intensity was gradually increased, following Borreani’s recommendations, progressing from static to dynamic exercises, from stable to unstable surfaces, and from eyes open to eyes closed	30 to 45 min	Exercises focused on the non-dominant leg. The protocol included static and dynamic balance exercises with bipedal and unipedal support, performed on stable and unstable surfaces, with eyes open and closed, incorporating tests such as the Y-Balance and Star Excursion. L	N/A	N/A
Lee, A. C., Sankaravel, M., & Chen, Z. X. (2023) [113]	3 times per week for 6 weeks	Training progression was key, incorporating a combination of postures with perturbations that varied in number, speed, and predictability (either constant or random)	30/45 min	(a) Tandem stance (b) Split squat stance (c) Single-leg stance (d) Bilateral jumping and landing (e) Single-leg jumping and landing	Not applicable	Not applicable
Pop, Nicolae & Ilisei, Irina. (2023) [114]	8 weeks × 3 sessions per week	Not applicable	Not Applicable	Not applicable	Not Applicable	N/A
Karkamandi, Fatemeh & Miri, Hadi & Letafatkar, Amir & Haghighi, Mina. (2022) [115]	9 weeks × 3 sessions per week	N/A	30/40 min	N/A	N/A	N/A
Cain, M. S., Ban, R. J., Chen, Y. P., Geil, M. D., Goerger, B. M., & Linens, S. W. (2020) [116]	3 sessions per week for 4 weeks	Not Applicable	Not Applicable	3 plantar flexion, dorsiflexion, inversion, and eversion exercises of the ankle using a resistance band. The group using the Biomechanical Ankle Platform System performed 5 trials of clockwise and counterclockwise rotations, changing direction every 10 s during each 40-s trial	3 sets × 10 rep	N/A
Kim, K. M., Estudillo-Martínez, M. D., Castellote-Caballero, Y., Estepa-Gallego, A., & Cruz-Díaz, D. (2021) [117]	Not applicable	Not applicable	Not applicable	Balance training group: supervised multimodal exercises addressing static and dynamic balance tasks. Stroboscopic + Balance training; Control group received no intervention	Not applicable	Not applicable
Sierra-Guzmán, R., Jiménez-Diaz, F., Ramírez, C., Esteban, P., & Abián-Vicén, J. (2018) [118]	3 sessions per week for 6 weeks	Intensity increased in the 4TH session	Not applicable	Week 1–3: One-legged stance cross-legged sway runner’s pose catching and throwing a volleyball against the wall Weeks 3–6: One-legged stance with eyes closed cross-legged sway with an elastic resistance band attached to the ankle runner’s pose with single-legged heel raises catching and throwing a tennis ball against the wall NVIB Group (no vibration): Trained on a BOSU (placed on the floor). VIB Group (with vibration): Trained on a vibration platform (Excel Pro Fitvibe), performing 4 exercises lasting 45 s each for 3 repetitions, with a 45-s rest between exercises. The difficulty of the exercises was increased in week 4. The vibration frequency was increased by 5 Hz every 2 weeks. The amplitude increased from 2 to 4 mm after the first week and remained at that level for the remainder of the study.	Not applicable	48 h between sets, 45 s between exercises
Chang, W., Chen, S., & Tsou, Y. (2021) [119]	6 weeks × week × 3 sessions	N/A	30 min	30 min/session: 5 min warm-up, 20 min exercise, 5 min cool-down). In weeks 1–3, 4 sets of 45 s/position with 40 s rest were performed, and in weeks 4–6, 5 sets of 45 s/position with 30 s rest. Group A worked on a vibration platform (5 Hz, 3 mm), Group B on a non-vibrating BOSU ball, and Group C continued their usual activity without intervention.	Not Applicable	Not applicable
Cruz-Diaz, D., Lomas-Vega, R., Osuna-Pérez, M. C., Contreras, F. H., & Martínez-Amat, A. (2015) [120]	3 sessions per week for 6 weeks	Not Applicable	Not Applicable	Balance training with 7 different activities, 3 sessions per week for 6 weeks control group underwent regular lower limb strength training only	Not applicable	Not applicable
Cain, M. S., Garceau, S. W., & Linens, S. W. (2017) [121]	3 times a week for 4 weeks	Not applicable	40 s	The intervention group performed a 4-week protocol using a biomechanical ankle platform system (BAPS), 3 times a week. Meanwhile, the control group received no intervention. Each session consisted of 5 trials of clockwise/counterclockwise rotations, alternating direction every 10 s during each 40-s trial.	4X10SEC	N/A
Hall, E. A., Chomistek, A. K., Kingma, J. J., & Docherty, C. L. (2018) [122]	3 times per week for 6 weeks	Moderate	20 min	Strength training: elastic band exercises for dorsiflexion, inversion, and eversion; plantar flexion on a step; PNF for slow inversion. Control group cycled for 20 min at moderate intensity.	N/A	Not applicable
Linens, S.W., Ross, S.E., & Arnold, B.L. (2016) [123]	3 times per week for 4 weeks	5 sets	40 s	Balance board group: 5 rotations (clockwise and counterclockwise) for 40 s each, with a 1-min rest between sets, 3 sessions per week, for 4 weeks. The control group received no intervention.	Not applicable	60 s
McKeon, P. O., Ingersoll, C. D., Kerrigan, D. C., Saliba, E., Bennett, B. C., & Hertel, J. (2008) [124]	3 times a week for 4 weeks	Not applicable	20 min	Progressive dynamic activities with 7 difficulty levels, where advancement to a new level depended on error-free execution of the previous one. Three types of progressive single-leg hops were included: (1) “hop to stabilization” in four directions (anterior/posterior, medial/lateral, anterolateral/posteromedial, and anteromedial/posterolateral), increasing the hop distance and difficulty (with or without hand support, and from platforms); (2) “hop to stabilization and reach,” which added a reaching component; and (3) “unanticipated hop to stabilization,” which involved random hops on a grid with decreasing reaction times and the incorporation of unstable surfaces at advanced levels. Additionally, single-leg balance was performed with eyes open and closed, both on firm ground and on a foam pad, and with medicine ball throws, gradually increasing the duration and complexity of the exercises.	Not Applicable	Not Applicable
Kim, K.-M., Estepa-Gallego, A., Estudillo-Martínez, M. D., Castellote-Caballero, Y., & Cruz-Díaz, D. (2022) [125]	2 times a week for 16 weeks	Not applicable	30–90 s	Consisting of 6 progressive exercises performed barefoot with the affected ankle. The training included: single-leg balance with arms crossed (floor, mat, Dynair, BOSU) for 30–90 s, single-leg ball toss/catch (10 repetitions), single-leg deadlift (10 reps with variations: hands on hips, with weight, 3-point reach), lateral jumps between cones, and forward-backward jumps (10 reps at distances of 30 cm, 45 cm, and 1 m), and random jumps in 4 directions (10 reps). Each exercise advanced through 4 stages, only after completing the previous level without errors. Complete circuit of the 6 exercises, mastering each level before progressing.	10 reps	N/A
Wright, C. J., Linens, S. W., & Cain, M. S. (2017) [126]	4 weeks × 2–4 sessions	4 levels	40 s	There were two protocols: Wobble Board (WB): 5 sets of 40-s rotations on an instability board (changing direction every 10 s), with 60 s of rest. Progression every 2–4 sessions based on control during gentle rotations, moving from level 1 to 5 (clockwise and counterclockwise).	5 sets of 40 s	60 s
Hajouj, E., Hadian, M. R., Mir, S. M., Talebian, S., & Ghazi, S. (2021) [127]	6 weeks × 2 sessions per week	Progressive between exercises	45/60 min	(1) Single-leg stance with eyes open. (2) Single-leg stance with eyes closed. (3) Single-leg stance with leg swing and eyes open. (4) Single-leg stance with leg swing and eyes closed. (5) Single-leg squat with eyes open and knee flexed to 30°. (6) Single-leg squat with eyes closed and 30° knee flexion. (7) Standing on a foam roll, barefoot, with double support. (8) Single-leg stance on a foam roll, barefoot. (9) Single-leg stance with leg swing on a foam roll, barefoot. (10) Walking forward on a foam roll with arms crossed, barefoot, and with double support. (11) Single-leg stance on a foam roll while throwing and catching a ball with the therapist. (12) Walking forward on a foam roll while throwing and catching a ball with the therapist.	Not Applicable	Not applicable
Shen, M., Che, S., Ye, D., Li, Y., Lin, F., & Zhang, Y. (2021) [128]	5 days × 4 weeks × 20 min	Differentiated by incline (0°, 5°, 10°, 15°)	Not applicable	Walking backward on a treadmill at different angles differentiated by incline (0°, 5°, 10°, 15°)	Not applicable	Not applicable

## 9. Discussion

This review highlights a problem common to many of the studies: the term ‘proprioceptive training’ is used to describe interventions that vary greatly from one another and are rarely linked to the sensory capacity implied by the concept. This label encompasses exercises on unstable surfaces [83], studies applying therapist-guided exercises to improve trunk control [75] or exercise programmes following injuries such as knee arthroplasty [89], This has caused the concept to lose precision and be applied to almost any task involving balance or body control, making it necessary to examine carefully how this type of training is interpreted.

When such heterogeneous interventions are given the same name, it is difficult to identify which mechanism explains the reported changes, as many of the described improvements may stem from motor learning, postural adjustments or increases in strength, rather than changes in sensory sensitivity. This confusion leads to conclusions that appear accurate without any measurements to support them, and it also limits clinical practice and research because professionals select exercises based on traditional criteria rather than defined somatosensory mechanisms. This situation makes it clear that a more precise use of the term ‘proprioceptive’ is needed to avoid confusing different abilities. The review was designed to clarify whether there is indeed any common feature that allows such diverse interventions to be termed proprioceptive, and to determine whether they share a principle that justifies their grouping. The gap between the physiological definition of proprioception—based on the detection of position, movement and force—and practice, which groups together motor tasks that rarely target these submodalities directly, made it necessary to assess whether the field is actually training proprioception or whether the term is used broadly to describe activities that primarily affect movement control. The aim was to provide a clearer basis for interpreting the available evidence. When examining the studies from a general perspective, a recurring pattern emerges, as most interventions labelled as proprioceptive focus on balance, stability or body control rather than the sensory signals specific to the detection of position, movement or force. A clear example can be seen in the work of Cruz-Diaz and Lomas-Vega [120], where improvements are reported following exercises combining mobility and stability without specific sensory measurements to confirm that there was a genuine change in proprioception; this suggests that much of these improvements can be explained by motor learning rather than by changes in receptor sensitivity. The findings also appear contradictory because the interventions use very different stimuli and yet reach similar conclusions regarding a supposed proprioceptive improvement, even though they are not actually measuring that capacity. Something similar occurs with Baltaci and Harput [77], who employ dynamic perturbations and challenges requiring rapid and coordinated motor responses, but subsequently interpret the changes as characteristic of proprioception even though the tests used assess motor performance rather than sensory submodalities. This pattern is repeated in various studies showing similar results even when they do not share the same type of stimulus.

Methodological inconsistency becomes evident when this literature is contrasted with the physiology of the somatosensory system, as proprioception requires the measurement of sensory errors, movement thresholds and force perception. Most studies do not target these submodalities in their interventions and use tests that incorporate coordination, strength or postural control, which allows any progress in movement to be interpreted as proprioceptive. This is clearly seen in the study by Lim and Cho where improvements occur without an intervention specifically targeting the sensory system, confirming that a person can improve their performance without proprioceptive sensitivity having changed directly. This distinction is important for understanding why the literature gives the impression that any change in movement is a proprioceptive effect when there are no measurements to support that claim.

The analysis also reveals recurring methodological weaknesses because many studies do not precisely define what they mean by proprioception nor explain how they plan to stimulate each sensory submodality. This leads to broad descriptions that do not make it clear whether the aim is to improve the detection of position, movement or force, or whether the intention is simply to increase stability. Some programmes alter the level of difficulty without clear criteria, as seen in the work by Harry-Leite and Paquete [111], where progressions depend more on the participant’s tolerance than on parameters related to sensory load. This method of dosing limits the interpretation of results because no relationship can be established between the magnitude of the stimulus, sensory fatigue or joint detection ability, making it difficult to assess whether the interventions actually target a proprioceptive mechanism or simply increase motor demand.

Heterogeneity is also evident in the exercises used, their duration, intensity and frequency, as each study employs different tasks yet reports similar conclusions. For example, in the work by Kim and Estudillo-Martínez [117], training was conducted on unstable surfaces with progression patterns that bear no relation to those of other authors who obtain similar results despite not sharing their approach. Added to this is a marked discrepancy between what is claimed to have been trained and what is actually measured, as many assessments are based on balance, stability or stance on a surface, as seen in studies using balance boards or platforms [85,90]. These measurements capture general motor performance, not the sensitivity of proprioceptive receptors. The absence of specific tests to detect sensory errors, such as movement thresholds or force perception, prevents us from establishing whether there was a real change in proprioception or whether the improvements reflect a better motor strategy or greater familiarity with the task.

Another frequently cited issue is the lack of control over the load, as none of the reviewed studies quantifies the magnitude of the proprioceptive stimulus or distinguishes between signals relating to joint velocity, tension or position [43,44,45]. Progressions are established based on motor difficulty without considering sensory parameters, and this prevents determining whether any specific pathway was stimulated. This lack of precision leads to many changes being interpreted as proprioceptive without any evidence to support this, and explains why the studies offer inconsistent conclusions.

Even with these limitations, the studies demonstrate beneficial changes in rehabilitation because programmes integrating movement control, stability and neuromuscular training improve mobility, reduce pain and increase confidence when performing daily activities in people with osteoarthritis or following joint replacement surgery. These results have clinical value, although they do not necessarily reflect a sensory change. The problem arises when they are labelled as ‘proprioceptive’, as two very different programmes may share the same name without sharing the same mechanism, which complicates communication between professionals and the interpretation of the literature. A clear example appears in the study by Moezy and Olyaei [78] where participants showed improvements in mobility and pain without movement detection or joint sensitivity being assessed. In the work by Huber and Roos [86], which evaluated stabilisation programmes following knee injury, progress was reported in tasks and clinical tests; however, the measurements focused on motor performance and general stability without quantifying sensory errors or parameters specific to proprioception, so it is not possible to state that the somatosensory system has been modified. Another representative case is that of Kaya and Guney-Deniz [93], where a programme labelled as proprioceptive yields improvements in pain and knee mobility, as well as greater confidence in performing daily activities. The figures reveal that the changes correspond to broad neuromuscular reorganisations rather than direct modifications of proprioception.

The reviewed evidence indicates that the term ‘proprioceptive training’ has been used so broadly that it has lost coherence with the physiology of the somatosensory system and with the variables actually assessed in the studies. A more rigorous way of using it would be to reserve it solely for interventions whose design, dosage and measurement explicitly focus on sensory submodalities such as the detection of position, movement or force, and which include tests capable of quantifying changes in those capabilities. In other cases, it is more honest to describe programmes according to their stated primary objective and what was actually measured—for example, whether the focus was on postural stability, coordination or movement organisation—rather than grouping all interventions under the same ‘proprioceptive’ label. This terminological precision would allow results to be interpreted with greater clarity and support interventions whose methodological rationale is consistent with what is actually stimulated and measured in each study.

### 9.1. Distinguishing True Proprioceptive Training from Sensorimotor Conditioning in Practice

The distinction advanced throughout this review can be operationalised, and it is worth illustrating it with concrete clinical cases rather than leaving it at the level of principle. Consider two interventions frequently reported under the same label after lateral ankle sprain. In the first, the patient performs single-leg stance on a foam pad with the eyes closed, progressing to a wobble board and then to hopping. In the second, the patient performs an ankle inversion-angle discrimination task on a device that presents a set of closely spaced inversion depths, judges which depth was reached on each trial, and receives knowledge of results, with the spacing between depths narrowed as accuracy improves. Both are commonly called proprioceptive training. Only the second manipulates a defined sensory variable, requires a perceptual judgement, and can demonstrate an improvement in discrimination acuity that is not reducible to strength, confidence or motor strategy. The first is postural-control training: valuable, evidence-based, and frequently the correct clinical choice, but its mechanism is not sensory refinement, and reporting it as proprioceptive obscures what actually produced the benefit.

The same contrast applies elsewhere. After anterior cruciate ligament reconstruction, unstable-surface squats with perturbations are neuromuscular conditioning, whereas knee joint-position matching at progressively finer target-angle differences with vision occluded and augmented feedback is proprioceptive training in the strict sense. In chronic neck pain, craniocervical flexion endurance work is motor-control training, whereas cervical repositioning practice against a laser target with error feedback trains position sense. In the upper limb after stroke, reaching practice towards functional targets is task-oriented motor training, whereas robotic arm-position matching with the affected limb, contrasted against the unaffected side, targets position sense directly. In each pair, the second intervention alone permits the inferential chain from sensory mechanism to sensory outcome that the label presupposes.

This is not an argument that sensorimotor conditioning is inferior or should be abandoned. It is an argument about attribution. Most of the interventions in Table 2 and Table 3 improve function, and the clinical decision to use them is usually well founded. What does not follow is the inference that they improved proprioception, because in the great majority of cases, proprioception was never measured, and where it was, the instrument was a balance index that cannot isolate it. Naming an intervention after a mechanism it has not been shown to engage is a claim about physiology, not a matter of terminological preference, and it has consequences: it inflates the apparent evidence base for sensory training, it prevents the accumulation of comparable data, and it leaves clinicians unable to select between interventions on mechanistic grounds.

### 9.2. Proprioception in Neurological Rehabilitation

The consequences of this conceptual imprecision are most acute in neurological rehabilitation, where sensory restoration is frequently the explicit therapeutic target rather than a presumed by-product of motor practice. Proprioceptive deficits affect approximately 50–64% of stroke survivors [32,33] and predict motor recovery, functional independence and discharge destination [32,34]. Decisively for the argument of this review, robotic assessment has shown that sensory and motor recovery after stroke follow partly independent trajectories: patients may regain motor function while position sense remains impaired, and the converse also occurs [35]. This dissociation is the strongest available empirical refutation of the assumption that motor improvement can be read as evidence of proprioceptive improvement. Where that assumption is embedded in the design of a trial, as it is in most of the studies retrieved here, the trial cannot answer the question it purports to answer.

Neurorehabilitation also illustrates what a coherent proprioceptive intervention looks like. Robotic position- and movement-matching paradigms specify the submodality, quantify error in degrees, and are sensitive to change independently of strength [34,35]. They are, at present, the clearest instantiation of the framework proposed in Figure 4, and their principal limitation is access rather than logic. The clinical challenge is to translate their measurement discipline into instruments that are affordable and deployable at the bedside without collapsing back into the coarse ordinal scales that dominate routine practice [34].

Two further strands of the stroke literature deserve explicit comment because they are often grouped with proprioceptive training. The first is goal-oriented, task-specific practice. Repetitive task training improves upper- and lower-limb function after stroke, with small to moderate effects, and is a cornerstone of contemporary practice [36]. Its mechanism, however, is the repetition of functionally meaningful motor sequences, not sensory discrimination; it belongs to the right-hand branch of the taxonomy in Figure 3. The productive question is not whether it competes with proprioceptive training but whether the two are complementary: sensory retraining may plausibly restore the afferent information on which task-specific practice depends, and combining them in a factorial design would be a far more informative trial than another comparison of an undifferentiated ‘proprioceptive’ programme against usual care.

The second strand is dual-task training. Cognitive–motor interference is substantial after stroke, with gait deteriorating measurably under concurrent cognitive load [37], and dual-task protocols improve gait and balance under dual-task conditions in randomised trials [38,129]. Dual-task work is highly relevant to proprioception because attentional load degrades the processing of afferent information, and because real-world ambulation is never a single-task condition, which is exactly the ecological objection raised in Section 6. Yet increasing cognitive demand is not the same as increasing sensory demand. A dual-task protocol that never manipulates joint angle, velocity or tension, and never asks the participant to judge them, trains the allocation of attention during movement, not proprioceptive acuity. Dual-tasking is best understood as a constraint to be manipulated within Step 3 of the proposed framework once a submodality has been targeted, rather than as a category of proprioceptive training in its own right.

### 9.3. Proprioception in Fibromyalgia and Chronic Pain

Chronic pain conditions raise a distinct and equally instructive problem. In fibromyalgia, postural instability, altered body perception and falls are well documented, and proprioceptive training has been incorporated into multidisciplinary protocols for patients presenting with imbalance, in combination with education, mindfulness and exercise training [31]. The interpretative difficulty is that the proprioceptive dysfunction here is unlikely to originate in the receptors. Central sensitisation, altered cortical body representation, pain-related attentional capture and fatigue can each degrade performance on any proprioceptive test without any peripheral afferent deficit. An elevated joint-position error in fibromyalgia is therefore genuinely ambiguous: it may reflect degraded afferent signalling, degraded central processing of an intact signal, or the confounding effect of pain on the response itself.

This ambiguity has a direct methodological implication that the field has largely ignored. If the deficit is central rather than peripheral, then an intervention targeting receptor stimulation is aimed at the wrong level of the system, and an improvement in position-matching error after treatment may reflect reduced pain or improved attention rather than restored proprioceptive acuity. Distinguishing these possibilities requires that pain intensity and attentional state be measured concurrently with the proprioceptive outcome and modelled as covariates, which is rarely done. Fibromyalgia thus makes explicit a requirement that applies across this literature: an intervention should be specified not only by the submodality it targets, but by the level of the system, whether peripheral, spinal or cortical, at which it claims to act. The framework proposed in Figure 4 is a necessary condition for coherent practice, not a sufficient one; where the dysfunction is centrally mediated, Step 1 must identify the level as well as the submodality.

### 9.4. Limitations and Future Directions

Despite the comprehensive scope of this narrative review, several limitations must be acknowledged. First, the non-systematic nature of the methodology may introduce selection bias, as the inclusion of studies was not governed by a pre-registered protocol or exhaustive systematic criteria. Although efforts were made to follow methodological recommendations for narrative reviews and to include relevant and representative literature, the possibility of omitting pertinent studies cannot be entirely excluded.

Second, the heterogeneity of the literature represents a major limitation not only of this review but of the field itself. The lack of consistent definitions, assessment methods, and intervention protocols across studies makes it difficult to establish clear comparisons or draw definitive conclusions. In particular, the frequent conflation of proprioception with broader sensorimotor or neuromuscular constructs complicates the interpretation of findings and limits the specificity of the conclusions presented.

Third, many of the studies included rely on assessment tools with limited ecological validity, often conducted under controlled laboratory conditions that do not accurately reflect real-world motor behaviour. This limitation restricts the ability to translate findings into applied settings, particularly in sports performance and rehabilitation contexts where dynamic and unpredictable environments are the norm.

Additionally, the absence of standardised operational definitions of proprioceptive training across the literature prevents the identification of specific mechanisms underlying observed adaptations. As a result, it remains unclear whether reported improvements are attributable to genuine changes in sensory discrimination processes or to broader motor adaptations such as strength, coordination, or motor learning.

### 9.5. Future Directions

Future research should prioritise the development of a clear and operational definition of proprioceptive training, grounded in the differentiation of its underlying submodalities (joint position sense, kinaesthesia, and force perception). Establishing such a framework would allow for more precise alignment between intervention design, targeted mechanisms, and outcome measures.

Moreover, there is a need for the development and validation of assessment tools with greater ecological validity, capable of capturing proprioceptive function within dynamic and context-specific environments. Integrating wearable technology, virtual reality, or task-specific testing paradigms may offer promising avenues to bridge the gap between laboratory measurements and real-world performance.

From an intervention perspective, future studies should aim to isolate and manipulate specific sensory components, rather than relying on multimodal protocols that obscure the mechanisms of adaptation. This includes designing interventions that explicitly target sensory discrimination processes and verifying their effects using modality-specific outcome measures.

Longitudinal and controlled experimental designs are also required to determine the dose-response relationship of proprioceptive stimuli and to establish progression criteria based on measurable changes in sensory function. Furthermore, research should consider population-specific responses, recognising that adaptations may differ across athletes, clinical populations, and individuals with varying levels of baseline proprioceptive function.

Finally, advancing the field will require a shift from descriptive and heterogeneous approaches towards mechanistically driven research, where interventions, measurements, and theoretical frameworks are coherently aligned. Only through this integration will it be possible to distinguish true proprioceptive adaptations from general sensorimotor improvements and to translate scientific evidence into effective clinical and sporting practices.

## 10. Conclusions

The review revealed that the use of the term ‘proprioceptive training’ lacks an operational definition compatible with the physiology of the somatosensory system and with the methods employed in the literature. The included protocols demonstrate conceptual and methodological heterogeneity that prevents linking the described benefits to changes in the detection of position, movement or force, making it more plausible to interpret them as general motor adaptations. The absence of explicit sensory models for criteria to dose stimuli and of measurements capable of assessing proprioceptive submodalities limits the validity of claims attributing improvements to a sensory mechanism. Based on this critical analysis, the term ‘proprioceptive’ should only be used when there is a clear methodological intention and appropriate instruments to measure the sensory capacity intended to be modified, whilst other interventions require description according to their motor purpose and the variables they actually quantify. Without this conceptual and methodological clarification, the efficacy attributed to proprioceptive training will remain difficult to interpret, and the field will continue to be plagued by confusion that affects both the comparison of studies and their clinical and sporting application.

## Figures and Tables

**Table 1:** Comparison of the main methods used to assess proprioception: targeted submodality, reported psychometric performance, use of control groups and ecological validity.

Assessment Method	Submodality Assessed	Reported Psychometric Performance (Statistical Results)	Control Groups in the Reviewed Evidence	Ecological Validity	Ref.
Active joint position reproduction (goniometry, inclinometry, isokinetic dynamometry)	Joint position sense	Absolute/constant error is the usual metric; reliability reported from poor to good and strongly protocol-dependent (target angle, number of trials, limb tested), which limits pooling across studies	Frequently absent; where present, usually a contralateral-limb or healthy between-group comparison rather than a randomised control	Low to moderate: seated, single plane, vision occluded	[6,43,45]
Passive joint position reproduction	Joint position sense	Comparable reliability to the active version with the motor-execution confound reduced; requires an isokinetic device, which restricts uptake	Often absent	Low	[43,44]
Threshold to detection of passive motion (TTDPM)	Kinaesthesia	Moderate to good reliability when angular velocity is standardised; results are highly sensitive to velocity, starting angle and attentional state	Often absent in intervention studies	Low: very slow passive motion bears little resemblance to gait or sport	[7,43,44]
Force reproduction and effort scaling (% of maximal voluntary contraction)	Force and effort sense	Performance depends critically on normalisation to maximal voluntary contraction and on fatigue control; the least standardised of the three submodalities, with no consensus protocol	Rarely reported	Low to moderate	[7,43]
Robotic manipulanda (position- and movement-matching tasks)	Joint position sense and kinaesthesia	Highest measurement precision; able to dissociate position sense from kinaesthesia and both from motor impairment, and validated against clinical scales in stroke cohorts	Healthy age- and sex-matched controls are standard in this literature	Low ecological validity but high internal validity	[35,42]
Movement-discrimination paradigms (AMEDA-type)	Kinaesthesia (discrimination)	Discriminates level of competitive success in athletes and relates to performance and injury history; scores reflect discrimination acuity rather than matching error	Between-group athlete comparisons rather than randomised controls	Moderate to high: active and weight-bearing	[30,48,49,50]
Stabilometric and posturographic platforms	Composite; no submodality isolated	Indices quantify postural control rather than proprioceptive acuity, so a change cannot be attributed to a sensory mechanism	Variable; frequently uncontrolled	Moderate	[26]
Clinical bedside scales (thumb localisation, Rivermead assessment)	Joint position sense (coarse)	Ordinal scoring with ceiling effects and low resolution; adequate for screening but insufficient to detect training-induced change	Common in neurological cohorts	Moderate	[34,43]
Cervical joint repositioning error	Joint position sense (cervical)	Widely used in neck-pain research; reliability degraded by pain, fatigue and head-movement strategy	Frequently symptomatic versus asymptomatic comparison	Moderate	[45]

Psychometric performance is summarised qualitatively as reported in the cited primary studies and reviews; coefficients are not directly comparable across studies because joint, protocol and population differ. AMEDA, Active Movement Extent Discrimination Apparatus; TTDPM, threshold to detection of passive motion.

## Data Availability

No new experimental data were created in this study. The review protocol and its supporting documentation are openly available in Figshare at https://doi.org/10.6084/m9.figshare.32101657.

## References

[1] Boddice R. (2023). Scientific and Medical Knowledge Production, 1796–1918: Volume III: Authority.

[2] Proske U., Gandevia S.C. (2012). The Proprioceptive Senses: Their Roles in Signaling Body Shape, Body Position and Movement, and Muscle Force. Physiol. Rev..

[3] Garbarino M.C., Pisani A., Biggiogera M., Mazzarello P. (2025). Camillo Golgi’s contributions to the anatomic basis of sensitivity in tendons. J. Neural Transm..

[4] Collins D.F., Binder M.D., Hirokawa N., Windhorst U. (2009). Proprioception: Role of Cutaneous Receptors. Encyclopedia of Neuroscience.

[5] Macefield V.G. (2021). The roles of mechanoreceptors in muscle and skin in human proprioception. Curr. Opin. Physiol..

[6] Goble D.J. (2010). Proprioceptive Acuity Assessment via Joint Position Matching: From Basic Science to General Practice. Phys. Ther..

[7] Héroux M.E., Butler A.A., Robertson L.S., Fisher G., Gandevia S.C. (2022). Proprioception: A new look at an old concept. J. Appl. Physiol..

[8] Sherrington C.S. (1911). The Integrative Action of the Nervous System.

[9] Shadrach J.L., Gomez-Frittelli J., Kaltschmidt J.A. (2021). Proprioception revisited: Where do we stand?. Curr. Opin. Physiol..

[10] Matthews P.B.C. (1988). Proprioceptors and their contribution to somatosensory mapping; complex messages require complex processing. Can. J. Physiol. Pharmacol..

[11] Marasco P.D., De Nooij J.C. (2023). Proprioception: A New Era Set in Motion by Emerging Genetic and Bionic Strategies?. Annu. Rev. Physiol..

[12] Lallemend F., Techameena P., Hadjab S. (2025). Functional and molecular insights into muscle proprioceptors. eLife.

[13] Missen K.J., Carpenter M.G., Assländer L. (2024). Velocity dependence of sensory reweighting in human balance control. J. Neurophysiol..

[14] Liu Y., Dong S., Wang Q., Liu Z., Song Q., Shen P. (2024). Deficits in proprioception and strength may contribute to the impaired postural stability among individuals with functional ankle instability. Front. Physiol..

[15] Witowski V., Lorbeer L., Schmid L., Wilhelmi B., Hoursch V.A., Carty M.J., Herr H.M., Blumer R., Sartori M., Yavuz U.Ş. (2026). Intramuscular tendon length in agonist–antagonist myoneural interface components in transtibial amputation: An anatomic study. J. Anat..

[16] Sun Y., Petrelli L., Fede C., Biz C., Incendi D., Porzionato A., Pirri C., Zhao X., Stecco C. (2025). Novel fascial mapping of muscle spindles distribution: Insights from a murine model study. Front. Physiol..

[17] Mao Y., Gao Z., Yang H., Song C. (2022). Influence of proprioceptive training based on ankle-foot robot on improving lower limbs function in patients after a stroke. Front. Neurorobot..

[18] Malwanage K.T., Liyanage E., Weerasinghe V., Antonypillai C., Nanayakkara I. (2024). A novel proprioceptive rehabilitation program: A pilot randomized controlled trail as an approach to address proprioceptive deficits in patients with diabetic polyneuropathy. PLoS ONE.

[19] Rezaee A., Daneshmandi H., Ramezanzade H., Mohammadzadeh S., Kurnaz M., Altınkök M. (2026). Improving coordination, proprioception, balance and motor proficiency in Down syndrome with developmental games. Exp. Physiol..

[20] Engin O., Kizilirmak Karatas A.S., Taspinar B., Taspinar F. (2026). Does the degree of stenosis affect cervical proprioception in patients with cervical pain?. J. Back. Musculoskelet. Rehabil..

[21] Sertic J.V.L., Kang J., MacKinnon C.D., Konczak J. (2025). Ankle proprioception and the relationship to rigidity in Parkinson’s disease. Clin. Neurophysiol..

[22] Vitharana T.N., King E., Moran K. (2024). Sensorimotor Dysfunction Following Anterior Cruciate Ligament Reconstruction- an Afferent Perspective: A Scoping Review. Int. J. Sports Phys. Ther..

[23] Ergen H.İ., Keskinbıçkı M.V., Öksüz Ç. (2024). The Effect of Proprioceptive Training on Hand Function and Activity Limitation After Open Carpal Tunnel Release Surgery: A Randomized Controlled Study. Arch. Phys. Med. Rehabil..

[24] Huang L., You G., Li M., Xia Z., Yang S., Zhou X., Shi H., Wang D., Zhang L. (2025). Outcomes of Proprioceptive Training on Recovery After Anterior Cruciate Ligament Reconstruction: A Systematic Review and Meta-analysis. Am. J. Phys. Med. Rehabil..

[25] Cantero-Téllez R., Algar L.A., Valdes K.A., Naughton N. (2022). Clinical effects of proprioceptive thumb exercise for individuals with carpometacarpal joint osteoarthritis: A randomized controlled trial. J. Hand Ther..

[26] Horváth Á., Ferentzi E., Schwartz K., Jacobs N., Meyns P., Köteles F. (2023). The measurement of proprioceptive accuracy: A systematic literature review. J. Sport Health Sci..

[27] Winter L., Huang Q., Sertic J.V.L., Konczak J. (2022). The Effectiveness of Proprioceptive Training for Improving Motor Performance and Motor Dysfunction: A Systematic Review. Front. Rehabil. Sci..

[28] Yılmaz O., Soylu Y., Erkmen N., Kaplan T., Batalik L. (2024). Effects of proprioceptive training on sports performance: A systematic review. BMC Sports Sci. Med. Rehabil..

[29] Arumugam A., Björklund M., Mikko S., Häger C.K. (2021). Effects of neuromuscular training on knee proprioception in individuals with anterior cruciate ligament injury: A systematic review and GRADE evidence synthesis. BMJ Open.

[30] Han J., Waddington G., Adams R., Anson J., Liu Y. (2016). Assessing proprioception: A critical review of methods. J. Sport Health Sci..

[31] Chiaramonte R., Bonfiglio M., Chisari S. (2019). Multidisciplinary protocol for the management of fibromyalgia associated with imbalance. Our experience and literature review. Rev. Assoc. Med. Bras..

[32] Yu Y., Chen Y., Lou T., Shen X. (2022). Correlation Between Proprioceptive Impairment and Motor Deficits After Stroke: A Meta-Analysis Review. Front. Neurol..

[33] Kessner S.S., Schlemm E., Cheng B., Bingel U., Fiehler J., Gerloff C., Thomalla G. (2019). Somatosensory Deficits After Ischemic Stroke: Time Course and Association with Infarct Location. Stroke.

[34] Findlater S.E., Dukelow S.P. (2017). Upper Extremity Proprioception After Stroke: Bridging the Gap Between Neuroscience and Rehabilitation. J. Mot. Behav..

[35] Semrau J.A., Herter T.M., Scott S.H., Dukelow S.P. (2015). Examining Differences in Patterns of Sensory and Motor Recovery After Stroke with Robotics. Stroke.

[36] French B., Thomas L.H., Coupe J., McMahon N.E., Connell L., Harrison J., Sutton C.J., Tishkovskaya S., Watkins C.L. (2016). Repetitive task training for improving functional ability after stroke. Cochrane Database Syst. Rev..

[37] Al-Yahya E., Dawes H., Smith L., Dennis A., Howells K., Cockburn J. (2011). Cognitive motor interference while walking: A systematic review and meta-analysis. Neurosci. Biobehav. Rev..

[38] Plummer P., Zukowski L.A., Feld J.A., Najafi B. (2022). Cognitive-motor dual-task gait training within 3 years after stroke: A randomized controlled trial. Physiother. Theory Pract..

[39] Freeman M.A., Wyke B. (1967). The innervation of the knee joint. An anatomical and histological study in the cat. J. Anat..

[40] Baethge C., Goldbeck-Wood S., Mertens S. (2019). SANRA—A scale for the quality assessment of narrative review articles. Res. Integr. Peer Rev..

[41] Kandel E.R. (2013). Principles of Neural Science.

[42] Hughes C.M.L., Tommasino P., Budhota A., Campolo D. (2015). Upper Extremity Proprioception in Healthy Aging and Stroke Populations, and the Effects of Therapist- and Robot-Based Rehabilitation Therapies on Proprioceptive Function. Front. Hum. Neurosci..

[43] Hillier S., Immink M., Thewlis D. (2015). Assessing Proprioception: A Systematic Review of Possibilities. Neurorehabilit. Neural Repair.

[44] Clark N.C., Röijezon U., Treleaven J. (2015). Proprioception in musculoskeletal rehabilitation. Part 2: Clinical assessment and intervention. Man. Ther..

[45] Röijezon U., Clark N.C., Treleaven J. (2015). Proprioception in musculoskeletal rehabilitation. Part 1: Basic science and principles of assessment and clinical interventions. Man. Ther..

[46] Justo-Cousiño L.A., Da Cuña-Carrera I., Alonso-Calvete A., González-González Y. (2024). Effect of Kinesio taping on wrist proprioception in healthy subjects: A randomized clinical trial. J. Hand Ther..

[47] Abbasi S., Hadian Rasanani M.R., Ghotbi N., Olyaei G.R., Bozorgmehr A., Rasouli O. (2020). Short-term effect of kinesiology taping on pain, functional disability and lumbar proprioception in individuals with nonspecific chronic low back pain: A double-blinded, randomized trial. Chiropr. Man. Ther..

[48] Han J., Anson J., Waddington G., Adams R., Liu Y. (2015). The Role of Ankle Proprioception for Balance Control in relation to Sports Performance and Injury. BioMed Res. Int..

[49] Han J., Waddington G., Anson J., Adams R. (2015). Level of competitive success achieved by elite athletes and multi-joint proprioceptive ability. J. Sci. Med. Sport.

[50] Long Z., Wang R., Han J., Waddington G., Adams R., Anson J. (2017). Optimizing ankle performance when taped: Effects of kinesiology and athletic taping on proprioception in full weight-bearing stance. J. Sci. Med. Sport.

[51] Cameron M.L., Adams R.D., Maher C.G. (2008). The effect of neoprene shorts on leg proprioception in Australian football players. J. Sci. Med. Sport.

[52] Rivera M.J., Winkelmann Z.K., Powden C.J., Games K.E. (2017). Proprioceptive Training for the Prevention of Ankle Sprains: An Evidence-Based Review. J. Athl. Train..

[53] Jenkins S. (2014). John R. Wooden, Stephen R. Covey and Servant Leadership. Int. J. Sports Sci. Coach..

[54] Shrier I., Impellizzeri F.M., Stovitz S.D. (2023). Prevention versus risk reduction or mitigation: Why create unnecessary battles?. J. Sci. Med. Sport.

[55] Alizamani S., Ghasemi G., Lenjan Nejadian S. (2023). Effects of eight week core stability training on stable- and unstable-surface on ankle muscular strength, proprioception, and dorsiflexion in athletes with chronic ankle instability. J. Bodyw. Mov. Ther..

[56] Alawna M., Mohamed A.A. (2020). Short-term and long-term effects of ankle joint taping and bandaging on balance, proprioception and vertical jump among volleyball players with chronic ankle instability. Phys. Ther. Sport.

[57] De Vries A.J., Van Den Akker-Scheek I., Diercks R.L., Zwerver J., Van Der Worp H. (2016). The effect of a patellar strap on knee joint proprioception in healthy participants and athletes with patellar tendinopathy. J. Sci. Med. Sport.

[58] Hosp S., Bottoni G., Heinrich D., Kofler P., Hasler M., Nachbauer W. (2015). A pilot study of the effect of Kinesiology tape on knee proprioception after physical activity in healthy women. J. Sci. Med. Sport.

[59] Yu R., Yang Z., Witchalls J., Adams R., Waddington G., Han J. (2021). Kinesiology tape length and ankle inversion proprioception at step-down landing in individuals with chronic ankle instability. J. Sci. Med. Sport.

[60] Chang L., Fu S., Wu S., Witchalls J., Adams R., Waddington G., Han J. (2022). Effects of graduated compression socks on ankle inversion proprioception of half-marathon runners at different running distances. J. Sci. Med. Sport.

[61] Alonso Martín A.H., Blanco R., Justo Cousiño L.A. (2019). Efectos del kinesiotape sobre el tono y la fuerza muscular. Revisión Sist. Sport Sci. J..

[62] Maldonado Lario A., Sancho García M.M., Mallor López E., Souto Ayerbe C., Vera Blasco N., Jubero Puntos A. (2021). Revisión bibliográfica sobre los efectos del kinesiotaping. Rev. Sanit. Investig..

[63] Otsuka S., Papadopoulos K., Bampouras T.M., Maestroni L. (2022). What is the effect of ankle disk training and taping on proprioception deficit after lateral ankle sprains among active populations?—A systematic review. J. Bodyw. Mov. Ther..

[64] Raymond J., Nicholson L.L., Hiller C.E., Refshauge K.M. (2012). The effect of ankle taping or bracing on proprioception in functional ankle instability: A systematic review and meta-analysis. J. Sci. Med. Sport.

[65] Biz C., Nicoletti P., Tomasin M., Bragazzi N.L., Di Rubbo G., Ruggieri P. (2022). Is Kinesio Taping Effective for Sport Performance and Ankle Function of Athletes with Chronic Ankle Instability (CAI)? A Systematic Review and Meta-Analysis. Medicina.

[66] Chamorro-Moriana G., Perez-Cabezas V., Benitez-Lugo M. (2024). Effectiveness of functional or biomechanical bandages with athletic taping and kinesiotaping in subjects with chronic ankle instability: A systematic review and meta-analysis. EFORT Open Rev..

[67] González Badillo J.J., Ribas-Serna J. (2002). Bases de la Programación del Entrenamiento de Fuerza.

[68] Callaghan M.J., Selfe J., McHenry A., Oldham J.A. (2008). Effects of patellar taping on knee joint proprioception in patients with patellofemoral pain syndrome. Man. Ther..

[69] Gabbett T.J., Oetter E. (2025). From Tissue to System: What Constitutes an Appropriate Response to Loading?. Sports Med..

[70] Riemann B.L., Lephart S.M. (2002). The sensorimotor system, part I: The physiologic basis of functional joint stability. J. Athl. Train..

[71] Al-Dadah O., Shepstone L., Donell S.T. (2011). Proprioception following partial meniscectomy in stable knees. Knee Surg. Sports Traumatol. Arthrosc..

[72] Ettinger L.R., Boucher A., Simonovich E. (2018). Patients with type 2 diabetes demonstrate proprioceptive deficit in the knee. World J. Diabetes.

[73] Kessner S.S., Bingel U., Thomalla G. (2016). Somatosensory deficits after stroke: A scoping review. Top. Stroke Rehabil..

[74] Duray M. (2018). The Investigation of the Effect of Proprioceptive Training on Balance in Patients with Chronic Neck Pain. Ağrı-J. Turk. Soc. Algol..

[75] Sipko T., Glibowski E., Kuczyński M. (2021). Acute effects of proprioceptive neuromuscular facilitation exercises on the postural strategy in patients with chronic low back pain. Complement. Ther. Clin. Pract..

[76] Pistone E., Laudani L., Camillieri G., Cagno A., Tomassi G., Macaluso A., Giombini A. (2016). Effects of early whole-body vibration treatment on knee neuromuscular function and postural control after anterior cruciate ligament reconstruction: A randomized controlled trial. J. Rehabil. Med..

[77] Baltaci G., Harput G., Haksever B., Ulusoy B., Ozer H. (2013). Comparison between Nintendo Wii Fit and conventional rehabilitation on functional performance outcomes after hamstring anterior cruciate ligament reconstruction: Prospective, randomized, controlled, double-blind clinical trial. Knee Surg. Sports Traumatol. Arthrosc..

[78] Moezy A., Olyaei G., Hadian M., Razi M., Faghihzadeh S. (2008). A comparative study of whole body vibration training and conventional training on knee proprioception and postural stability after anterior cruciate ligament reconstruction. Br. J. Sports Med..

[79] Akbari A., Ghiasi F., Mir M., Hosseinifar M. (2015). The Effects of Balance Training on Static and Dynamic Postural Stability Indices After Acute ACL Reconstruction. Glob. J. Health Sci..

[80] Da Silva Boitrago M.V., De Mello N.N., Barin F.R., Júnior P.L., De Souza Borges J.H., Oliveira M. (2021). Effects of proprioceptive exercises and strengthening on pain and functionality for patellofemoral pain syndrome in women: A randomized controlled trial. J. Clin. Orthop. Trauma..

[81] Zarei H., Norasteh A.A., Lieberman L.J., Ertel M.W., Brian A. (2023). Effects of proprioception and core stability training on gait parameters of deaf adolescents: A randomized controlled trial. Sci. Rep..

[82] Cooper R.L., Taylor N.F., Feller J.A. (2005). A Randomised Controlled Trial of Proprioceptive and Balance Training after Surgical Reconstruction of the Anterior Cruciate Ligament. Res. Sports Med..

[83] Vathrakokilis K., Malliou P., Gioftsidou A., Beneka A., Godolias G. (2008). Effects of a balance training protocol on knee joint proprioception after anterior cruciate ligament reconstruction. J. Back. Musculoskelet. Rehabil..

[84] Bitterli R., Sieben J.M., Hartmann M., De Bruin E.D. (2011). Pre-Surgical Sensorimotor Training for Patients Undergoing Total Hip Replacement: A Randomised Controlled Trial. Int. J. Sports Med..

[85] Gstoettner M., Raschner C., Dirnberger E., Leimser H., Krismer M. (2011). Preoperative proprioceptive training in patients with total knee arthroplasty. Knee.

[86] Huber E.O., Roos E.M., Meichtry A., De Bie R.A., Bischoff-Ferrari H.A. (2015). Effect of preoperative neuromuscular training (NEMEX-TJR) on functional outcome after total knee replacement: An assessor-blinded randomized controlled trial. BMC Musculoskelet. Disord..

[87] Jogi P., Overend T.J., Spaulding S.J., Zecevic A., Kramer J.F. (2015). Effectiveness of balance exercises in the acute post-operative phase following total hip and knee arthroplasty: A randomized clinical trial. SAGE Open Med..

[88] Liao C.D., Liou T.H., Huang Y.Y., Huang Y.C. (2013). Effects of balance training on functional outcome after total knee replacement in patients with knee osteoarthritis: A randomized controlled trial. Clin. Rehabil..

[89] Piva S.R., Gil A.B., Almeida G.J.M., DiGioia A.M., Levison T.J., Fitzgerald G.K. (2010). A Balance Exercise Program Appears to Improve Function for Patients with Total Knee Arthroplasty: A Randomized Clinical Trial. Phys. Ther..

[90] Villadsen A., Overgaard S., Holsgaard-Larsen A., Christensen R., Roos E.M. (2014). Postoperative effects of neuromuscular exercise prior to hip or knee arthroplasty: A randomised controlled trial. Ann. Rheum. Dis..

[91] Ordahan B., Küçükşen S., Tuncay İ., Sallı A., Uǧurlu H. (2015). The effect of proprioception exercises on functional status in patients with anterior cruciate ligament reconstruction. J. Back. Musculoskelet. Rehabil..

[92] Risberg M.A., Holm I., Myklebust G., Engebretsen L. (2007). Neuromuscular Training Versus Strength Training During First 6 Months After Anterior Cruciate Ligament Reconstruction: A Randomized Clinical Trial. Phys. Ther..

[93] Kaya D., Guney-Deniz H., Sayaca C., Calik M., Doral M.N. (2019). Effects on Lower Extremity Neuromuscular Control Exercises on Knee Proprioception, Muscle Strength, and Functional Level in Patients with ACL Reconstruction. BioMed Res. Int..

[94] Zheng Q.-y., Sun J.-n., Wang R.-s., Ma Y.-r., Chen P. (2025). Does proprioceptive training improve joint function and psychological readiness in patients after anterior cruciate ligament reconstruction? A randomized controlled trial. BMC Musculoskelet. Disord..

[95] Bąkowski P., Ciemniewska-Gorzela K., Bąkowska-Żywicka K., Stołowski Ł., Piontek T. (2021). Similar Outcomes and Satisfaction of the Proprioceptive versus Standard Training on the Knee Function and Proprioception, Following the Anterior Cruciate Ligament Reconstruction. Appl. Sci..

[96] Dragičević-Cvjetković D., Erceg-Rukavina T., Nikolić S. (2021). Proprioception recovery after anterior cruciate ligament reconstruction: Isokinetic versus dynamic exercises. Scr. Med..

[97] Rexha E. (2024). Use of proprioception during knee rehabilitation after anterior cruciate ligament reconstruction. J. Orthop..

[98] Lim J.M., Cho J.J., Kim T.Y., Yoon B.C. (2019). Isokinetic knee strength and proprioception before and after anterior cruciate ligament reconstruction: A comparison between home-based and supervised rehabilitation. J. Back. Musculoskelet. Rehabil..

[99] Bouisset S., Do M.C. (2008). Posture, dynamic stability, and voluntary movement. Neurophysiol. Clin. Neurophysiol..

[100] Sayyadi P., Minoonejad H., Seidi F., Shikhhoseini R., Arghadeh R. (2024). The effectiveness of fatigue on repositioning sense of lower extremities: Systematic review and meta-analysis. BMC Sports Sci. Med. Rehabil..

[101] Hadjisavvas S., Efstathiou M.A., Themistocleous I.C., Daskalaki K., Malliou P., Giannaki C.D., Lewis J., Stefanakis M. (2024). Effect of concentric exercise-induced fatigue on proprioception, motor control and performance of the upper limb in handball players. PLoS ONE.

[102] Seitz A.M., Murrmann M., Ignatius A., Dürselen L., Friemert B., von Lübken F. (2021). Neuromapping of the Capsuloligamentous Knee Joint Structures. Arthrosc. Sports Med. Rehabil..

[103] Gokeler A., Neuhaus D., Benjaminse A., Grooms D.R., Baumeister J. (2019). Principles of Motor Learning to Support Neuroplasticity After ACL Injury: Implications for Optimizing Performance and Reducing Risk of Second ACL Injury. Sports Med..

[104] Wolpert D.M., Diedrichsen J., Flanagan J.R. (2011). Principles of sensorimotor learning. Nat. Rev. Neurosci..

[105] Grooms D.R., Kiefer A.W., Riley M.A., Ellis J.D., Thomas S., Kitchen K., DiCesare C.A., Bonnette S., Gadd B., Foss K.D. (2018). Brain-Behavior Mechanisms for the Transfer of Neuromuscular Training Adaptions to Simulated Sport: Initial Findings from the Train the Brain Project. J. Sport Rehabil..

[106] Grooms D.R., Chaput M., Simon J.E., Criss C.R., Myer G.D., Diekfuss J.A. (2023). Combining Neurocognitive and Functional Tests to Improve Return-to-Sport Decisions Following ACL Reconstruction. J. Orthop. Sports Phys. Ther..

[107] Antohea B.A., Rață M., Rață B.C., Rață G. (2023). Proprioceptive exercises and their role in improving static and dynamic joint stability in ankle sprains in handball players. Sci. Sports.

[108] Ghaderi M., Letafatkar A., Almonroeder T.G., Keyhani S. (2020). Neuromuscular training improves knee proprioception in athletes with a history of anterior cruciate ligament reconstruction: A randomized controlled trial. Clin. Biomech..

[109] Gidu D.V., Badau D., Stoica M., Aron A., Focan G., Monea D., Stoica A.M., Calota N.D. (2022). The Effects of Proprioceptive Training on Balance, Strength, Agility and Dribbling in Adolescent Male Soccer Players. Int. J. Environ. Res. Public Health.

[110] Souglis A.G., Travlos A.K., Andronikos G. (2023). The effect of proprioceptive training on technical soccer skills in female soccer. Int. J. Sports Sci. Coach..

[111] Harry-Leite P., Paquete M., Teixeira J., Santos M., Sousa J., Fraiz-Brea J.A., Ribeiro F. (2022). Acute Impact of Proprioceptive Exercise on Proprioception and Balance in Athletes. Appl. Sci..

[112] Antohe B.A., Panaet E.A. (2024). The Effects of Proprioceptive Exercises on Postural Control in Handball Players with Chronic Ankle Instability—A Non-Randomized Control Trial. Sports.

[113] Lee A.C., Sankaravel M., Chen Z.X. (2023). The Effectiveness of Six-Week Balance Training with Perturbation Intervention in Enhancing Dynamic Balance of Basketball Players. Phys. Educ. Theory Methodol..

[114] Pop N.H., Ilisei I. (2022). Enhacement of swimming kinematics and performance through proprioception. Stud. Univ. Babeş-Bolyai Educ. Artis. Gymnast..

[115] Karkamandi F., Miri H., Letafatkar A., Haghighi M. (2021). Compare the effect of motor control exercises and PNF exercises on postural control strength endurance and proprioception in women with chronic nonspecific low back pain. Sport Sci. Health Res..

[116] Cain M.S., Ban R.J., Chen Y.P., Geil M.D., Goerger B.M., Linens S.W. (2020). Four-Week Ankle-Rehabilitation Programs in Adolescent Athletes with Chronic Ankle Instability. J. Athl. Train..

[117] Kim K.M., Estudillo-Martínez M.D., Castellote-Caballero Y., Estepa-Gallego A., Cruz-Díaz D. (2021). Short-Term Effects of Balance Training with Stroboscopic Vision for Patients with Chronic Ankle Instability: A Single-Blinded Randomized Controlled Trial. Int. J. Environ. Res. Public Health.

[118] Sierra-Guzmán R., Jiménez-Diaz F., Ramírez C., Esteban P., Abián-Vicén J. (2018). Whole-Body–Vibration Training and Balance in Recreational Athletes with Chronic Ankle Instability. J. Athl. Train..

[119] Chang W.D., Chen S., Tsou Y.A. (2021). Effects of Whole-Body Vibration and Balance Training on Female Athletes with Chronic Ankle Instability. J. Clin. Med..

[120] Cruz-Diaz D., Lomas-Vega R., Osuna-Pérez M., Contreras F., Martínez-Amat A. (2015). Effects of 6 Weeks of Balance Training on Chronic Ankle Instability in Athletes: A Randomized Controlled Trial. Int. J. Sports Med..

[121] Cain M.S., Garceau S.W., Linens S.W. (2017). Effects of a 4-Week Biomechanical Ankle Platform System Protocol on Balance in High School Athletes with Chronic Ankle Instability. J. Sport Rehabil..

[122] Hall E.A., Chomistek A.K., Kingma J.J., Docherty C.L. (2018). Balance- and Strength-Training Protocols to Improve Chronic Ankle Instability Deficits, Part I: Assessing Clinical Outcome Measures. J. Athl. Train..

[123] Linens S.W., Ross S.E., Arnold B.L. (2016). Wobble Board Rehabilitation for Improving Balance in Ankles with Chronic Instability. Clin. J. Sport Med..

[124] Mckeon P.O., Ingersoll C.D., Kerrigan D.C., Saliba E., Bennett B.C., Hertel J. (2008). Balance Training Improves Function and Postural Control in Those with Chronic Ankle Instability. Med. Sci. Sports Exerc..

[125] Kim K.M., Estepa-Gallego A., Estudillo-Martínez M.D., Castellote-Caballero Y., Cruz-Díaz D. (2022). Comparative Effects of Neuromuscular- and Strength-Training Protocols on Pathomechanical, Sensory-Perceptual, and Motor-Behavioral Impairments in Patients with Chronic Ankle Instability: Randomized Controlled Trial. Healthcare.

[126] Wright C.J., Linens S.W., Cain M.S. (2017). A Randomized Controlled Trial Comparing Rehabilitation Efficacy in Chronic Ankle Instability. J. Sport Rehabil..

[127] Hajouj E., Hadian M.R., Mir S.M., Talebian S., Ghazi S. (2021). Effects of Innovative Aquatic Proprioceptive Training on Knee Proprioception in Athletes with Anterior Cruciate Ligament Reconstruction: A Randomized Controlled Trial. Arch. Bone Jt. Surg..

[128] Shen M., Che S., Ye D., Li Y., Lin F., Zhang Y. (2021). Effects of backward walking on knee proprioception after ACL reconstruction. Physiother. Theory Pract..

[129] Baek C.Y., Chang W.N., Park B.Y., Lee K.B., Kang K.Y., Choi M.R. (2021). Effects of Dual-Task Gait Treadmill Training on Gait Ability, Dual-Task Interference, and Fall Efficacy in People with Stroke: A Randomized Controlled Trial. Phys. Ther..

